# Climate Change Predicted to Trigger an Upward Altitudinal Range Shift and Boost the Abundance of a Montane Rock Face Specialist, the Wallcreeper (*Tichodroma muraria*)

**DOI:** 10.1002/ece3.73963

**Published:** 2026-07-19

**Authors:** Célestin Luisier, Sergio Vignali, Veronika Braunisch, Arnaud G. Barras, Marc Kéry, Raphaël Arlettaz, Ian J. Ausprey

**Affiliations:** ^1^ Division of Conservation Biology, Institute of Ecology and Evolution University of Bern Bern Switzerland; ^2^ Department of Forest Nature Conservation Forest Research Institute of Baden‐Wuerttemberg Freiburg Germany; ^3^ Swiss Ornithological Institute Sempach Switzerland; ^4^ Department of Ecology & Conservation Biology Texas A&M University College Station Texas USA

**Keywords:** alpine, altitudinal migrant, annual life cycle, climate, elevation shift, rock face biodiversity, species distribution model

## Abstract

Montane species are predicted to respond to climate change by moving upslope, particularly high elevation specialists with thermal niches adapted to cold environments. Unfortunately, the accuracy of predictive species distribution models is often reduced by (1) the challenge of accounting for changes in habitat and resource availability and (2) the inability to account for complex life history strategies. Here we use the Wallcreeper ( *Tichodroma muraria* ) as a model species for predicting the response of high elevation specialists to climate change, leveraging unique aspects of its ecology as an obligate cliff specialist and altitudinal migrant. Using species distribution models based on citizen science data collected across Switzerland and intensive field surveys of abundance within its core range, we predict future range shifts and overlap among life cycle periods under different climate change scenarios. Principal environmental predictors varied among seasons, with temperature being more important during the winter. While the breeding range was predicted to shift upwards with little change in overall spatial extent, the overwintering range was predicted to expand upslope by up to 244%, leading to a 123% increase in seasonal overlap. Abundance models showed similar results while also providing the first robust density estimates for the species in Switzerland. In contrast to past studies of alpine biota that predict severe range contractions due to climate change, sufficient high elevation habitat exists in Switzerland to allow the Wallcreeper to shift and expand its range upslope, especially in the overwintering period when cold thermal constraints are relaxed. However, most of the current Wallcreeper distribution in Europe lies in mountain ranges likely to be outside of its breeding thermal range in the future, stressing the importance of the Swiss Alps as a stronghold for this species and alpine diversity in an era of rapidly warming temperatures.

## Graphical abstract

The distributional range of the Wallcreeper in Switzerland is predicted to greatly expand with global warming, primarily due to expansion of suitable conditions during the winter.

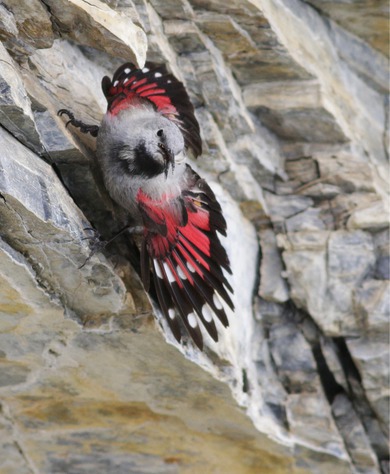

## 1 Introduction

Human‐induced climate change is predicted to impact biodiversity and ecosystem services through multiple phenomena, including increases in ambient air temperature, changes in precipitation regimes, and more frequent and pronounced extreme weather events such as flooding, drought, or landslides (Beniston 2003; Sala et al. 2000; Weiskopf et al. 2020). High latitude regions and mountain ranges appear particularly vulnerable because they are warming faster, on average, compared to other biomes (Beniston 2003; Pepin et al. 2015). For example, ambient temperatures in the Swiss Alps have already increased by 2°C since the industrial revolution and are expected to rise an additional 4.5°C by 2100 under the most extreme climate change scenarios (CH2018 2018; Kotlarski et al. 2023).

Mountains are also home to many cold‐adapted species that may be extremely sensitive to climate change due to their high degree of specialization, with short breeding periods and cold‐oriented thermal niches (Jiguet et al. 2010; Stephens et al. 2016; Tayleur et al. 2016). Given that birds are excellent dispersers, they are assumed to track their thermal niche by moving northward or upslope, and cold adapted montane species throughout the world seem to track temperatures by moving to higher elevations (Freeman et al. 2021; Tayleur et al. 2016; Tingley et al. 2009). The consequences of such upward movements often result in range contraction and isolation depending on the shape of mountains, phenological mismatches with prey, and local extinctions (Brambilla et al. 2017; Reif and Flousek 2012; Scridel et al. 2018; Socolar et al. 2017).

Species distribution models (SDMs) are often used to predict future range shifts by combining data on contemporary species occurrences, key environmental drivers, and future climate scenarios (Guisan et al. 2017). However, ecological complexity, such as food resources, breeding site availability, and potential interactions, often reduces the reliability of their results. First, it is often assumed that the availability and quality of ecological resources will remain similar under future conditions. Such assumptions ignore unknown factors that may further impact habitat quality and prey supply, notably new land‐use changes and altered species interactions (Pellissier et al. 2018). Second, studies using SDMs usually only focus on the breeding period (e.g., Barras, Braunisch, and Arlettaz 2021; Barras, Niffenegger, et al. 2021; Brambilla et al. 2022) or include the entire range of a species irrespective of stages in its annual life cycle (Guisan et al. 2017). Studies that account for specific annual stages are scarce (e.g., de Gabriel Hernando et al. 2021) even though considering the entire annual life cycle is critical for understanding distributions and population dynamics (e.g., Buechley et al. 2021; Sergio et al. 2019; Swift et al. 2020). For temperate resident species, the cold overwintering period is thought to be of particular importance (Bühler et al. 2023), especially for high elevation specialists given that they face extreme overwintering conditions that likely impact their fitness (Barçante et al. 2017; Robinson et al. 2007; Swanson 2002). Many of these species track their niche in winter by performing altitudinal migrations between breeding and overwintering areas that provide sufficient food resources and less harsh climatic conditions (Hsiung et al. 2018; Pageau et al. 2020). In the Alps, this mainly results in partial altitudinal migration, with some individuals staying in the surroundings of their breeding sites, but often at lower altitude, and some migrating latitudinally a few hundred kilometers to southern Europe (Barçante et al. 2017; Glutz von Blotzheim and Bauer 1993; Williamson and Witt 2021). Finally, while most SDMs on high elevation specialists' species only take distribution into account (e.g., Brambilla et al. 2022), including abundances can predict habitat suitability more accurately than simple occurrence probability and improve biological realism when defining important areas for conservation (Barras, Braunisch, and Arlettaz 2021; Johnston et al. 2015; Renwick et al. 2012).

For some partially‐migratory montane species, it has even been suggested that the possible negative effects of climate change during the breeding period could be partly compensated by positive effects experienced during the overwintering period as ambient temperatures become less extreme and reductions in snow cover enhance access to food (de Gabriel Hernando et al. 2021; but see Resano‐Mayor et al. 2019; Barras et al. 2020; Barras, Braunisch, and Arlettaz 2021; Barras, Niffenegger, et al. 2021). Moreover, as some high elevation specialists overwinter in lower mountain zones, their suitable overwintering area could expand upslope with rising temperature (Elsen and Tingley 2015), as has been observed latitudinally for some species (Maclean et al. 2008). However, information on interactions between breeding and non‐breeding periods remains limited (Rushing et al. 2020). Embracing an annual life cycle approach by considering the most critical periods of the year, especially for montane species, could help to make more realistic predictions for current and future distributions (de Gabriel Hernando et al. 2021).

In this study, we use the Wallcreeper as a model species for predicting the year‐round effects of climate change on high elevation montane species. The Wallcreeper is a small insectivorous short‐distance altitudinal migrant that breeds and forages exclusively on rock faces and is particularly suited for addressing this question for multiple reasons (Barçante et al. 2017; Löhrl 1976). First, the Wallcreeper's altitudinal migration pattern is representative of other palearctic high elevation specialists (Glutz von Blotzheim and Bauer 1993). Second, rock faces are considered priority habitats for research, as they represent fragile ecosystems experiencing increasing human activity (e.g., intensification of rock climbing) while harboring unique biodiversity that includes numerous species of plants, insects, bats and birds (Glutz von Blotzheim and Bauer 1993; Larson et al. 2000; March‐Salas et al. 2023). Third, the species' exclusive use of cliff habitat allows us to control for spatial and temporal habitat change that might confound the effects of climate change. For example, most other high elevation specialists forage on the ground, and snow cover is often a limiting factor that proves difficult to model accurately (Barras et al. 2020; Glutz von Blotzheim and Bauer 1993; Hsiung et al. 2018; Resano‐Mayor et al. 2019). Snow is rarely present on vertical rock faces, which eliminates this potentially confounding variable. Likewise, altitudinal migrants often use breeding and wintering habitats that are similar but still vary in structure and size (Malfasi and Cannone 2020). Rock faces used by Wallcreepers throughout the year provide extremely similar habitats that are highly unlikely to change in size and structure (Glutz von Blotzheim and Bauer 1993; Hauri 1970; Hauri 1978). Finally, the Wallcreeper defends both a fixed breeding and wintering territory, meaning that it rarely moves out of a small area within each season (Glutz von Blotzheim and Bauer 1993; Löhrl 1976; Luisier et al. 2022). Such spatial consistency should allow for better predictions in SDMs.

The overarching goal of this study is to predict the current and future breeding and winter distributions of the Wallcreeper under different climate scenarios by using species distribution models that integrate available occurrence data as well as intensive field surveys of abundance. Specifically, our objectives were to (1) develop breeding and overwintering SDMs based upon key environmental predictors including topography, climate, geology, land cover, rock face characteristics and connectivity, (2) quantify the population size of the species for a large part of its core European range using intensive field sampling of abundances, (3) predict future distributions and abundance of the species based on the most recent climate models and assess resulting changes during both breeding and overwintering periods, including spatial overlap, and (4) use these results to inform potential conservation measures regarding the sensitivity to climate change for this high elevation specialist and rock face habitats in general.

Overall, we hypothesized that climate variables would largely explain current and future distributions for the species. Specifically, we predicted that (1) range expansion during the over‐wintering season stemming from projected warmer temperatures would lead to increased inter‐seasonal range overlap, (2) such range expansion and overlap would be more pronounced for the most extreme climate change scenarios, and (3) incorporating abundances from field surveys would provide more precise estimates of current and projected population size.

## 2 Methods

### 2.1 Study Species

**FIGURE 1:**
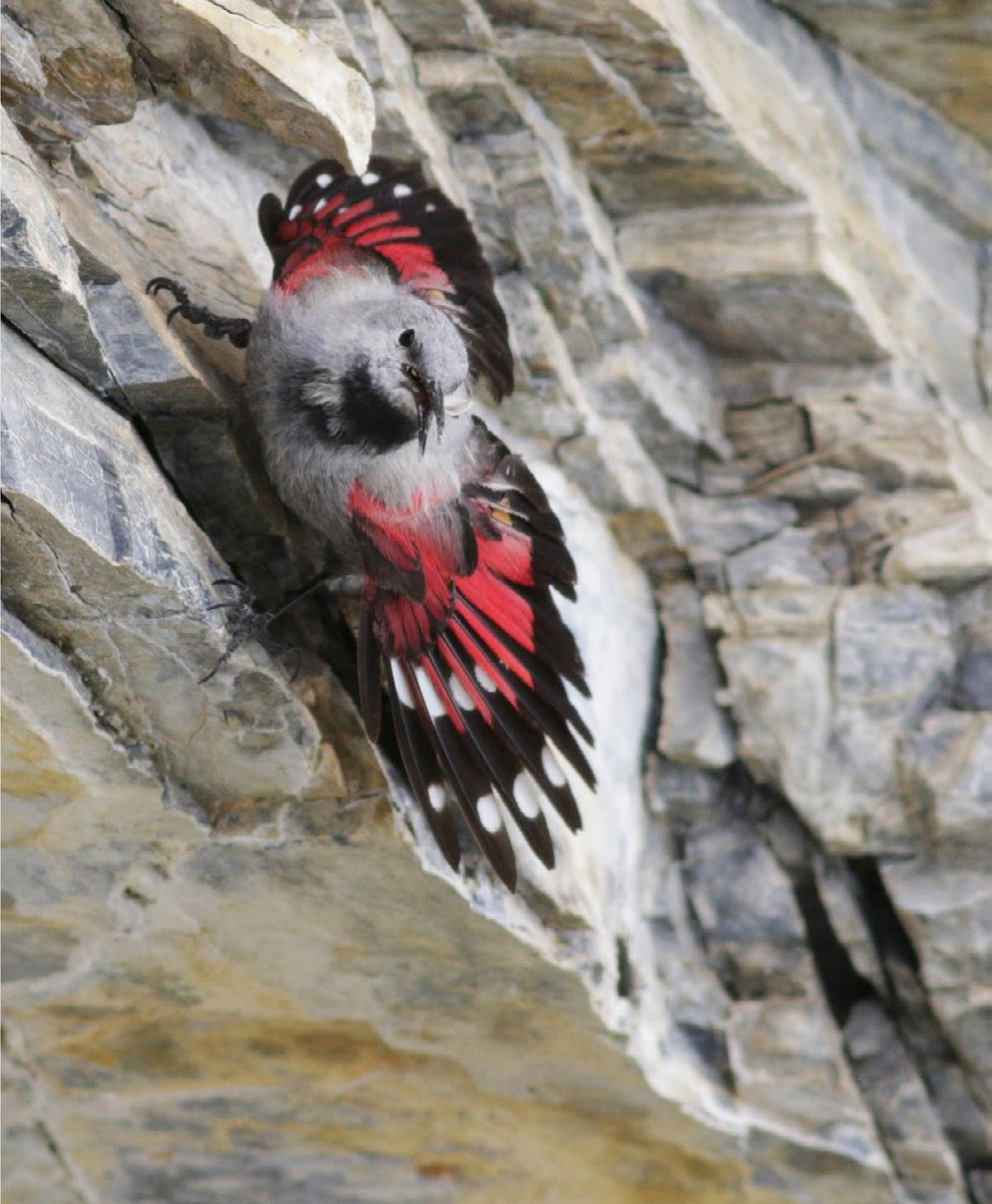
Wallcreeper ( *Tichodroma muraria* ) in Valais, Switzerland. Photo: Célestin Luisier.

The Wallcreeper is a small rock face obligate passerine occurring across high elevation mountains between 30° and 50° N from Spain to China (Figure 1). It breeds mainly in alpine habitats up to the nival belt, in addition to some medium to low elevation areas with cool microclimates, such as in gorges (Glutz von Blotzheim and Bauer 1993; Löhrl 1976; Luisier et al. 2020). The species arrives at breeding sites between March and May, disperses between July and September after the conclusion of reproduction, and arrives at wintering sites between late September and October (Löhrl 1976). The species specializes exclusively on cliffs characterized by numerous cracks, moderate vegetation coverage, high variability of exposure, and, to a limited extent, access to running water (Luisier et al. 2020; Saniga 1995, 2004, 2009).

While breeding densities in Europe are highest in alpine areas of temperate mountains such as the Alps and Pyrenees, the Wallcreeper's wintering population strongholds in western Europe are in medium altitude mountainous areas, such as large inner alpine valleys down to the Mediterranean coast (Maumary et al. 2007; Prampart 2021). Across Europe, including Switzerland, population trends during both breeding and wintering periods are virtually unknown, as the species is difficult to monitor. Although some studies have estimated minor distributional range contractions (7%–12%) or predict a short‐term loss of suitable habitat in southwest Europe, there is not sufficient evidence to detect or predict population trends (de Gabriel Hernando et al. 2021; Keller et al. 2020; Scridel et al. 2017). However, like other high elevation specialists, the species is predicted to be sensitive to climate change (Keller et al. 2020; Knaus et al. 2018).

### 2.2 Distribution Models for Switzerland

#### 2.2.1 Study Area

We constructed separate distribution models for the Wallcreeper during the breeding and wintering periods. We defined our study area as all rock faces included in the Swisstopo swissTLM3D geospatial layer, plus a 150 m buffer (Swisstopo 2022). For the breeding period, we further restricted our analysis to rock faces in the Jura mountains and the Alps, following the biogeographical regions of Switzerland (Gonseth and Sartori 2022), given that the breeding population is strictly constrained to high elevation mountainous areas.

#### 2.2.2 Presence Data

Wallcreeper presence data were extracted from the Swiss Ornithological Institute (SOI) database (ornitho.ch) for the entirety of Switzerland. Most of the data were collected by volunteer observers on an opportunistic basis, with additional records contributed by the Swiss monitoring of common breeding birds scheme (MHB) and other similar programs organized by the SOI (Knaus et al. 2018; Locher and Wezemael 2014; Schmid et al. 2004). The data were checked by SOI experts according to standard protocols (Schmid et al. 2023). We then split the dataset into the wintering period (November to February) and the breeding period (May to mid‐July), with the addition of records in April and mid‐July to August that were likely to be breeding observations (e.g., singing individuals, food provisioning activity, etc.). We retained observations only if they were collected (1) with high geographical accuracy (≤ 100 m), (2) within the reference study area, and (3) for the years 2010–2022 (see Methods S1 and Figure S1 for details on our data filtering protocol).

Because the data were largely collected by citizen scientists, they were likely biased in favor of popular bird watching locations (Kramer‐Schadt et al. 2013), particularly at low elevations during the winter. For the wintering period, we thus employed a spatial thinning procedure with a minimum distance of 200 m (Aiello‐Lammens et al. 2015), which corresponded to the expected distance between the centroids of individual territories in good quality habitat as estimated by Luisier et al. (2022). For the breeding period, we used a target group approach which acts only on the background points (Barber et al. 2022) (see Methods S3 for details). This resulted in a final sample size of 760 observations for the wintering period and 1497 observations for the breeding period.

#### 2.2.3 Environmental Predictor Variables

We used 28 environmental predictors hypothesized to influence Wallcreeper distribution. These were grouped into six categories: topography, climate, geology, land cover, rock face characteristics and connectivity (Table 1; See Methods S4 for predictors' calculation). All predictors were prepared as raster maps with a 25 m spatial resolution. Variables then were calculated as continuous percentages or averages within a 100 m radius circular moving window centered on the focal cell in the whole country (package *terra* (Hijmans 2022)). This 100 m radius corresponded to minimal spatial accuracy of the bird observations and allowed us to both consider the conditions within a territory and account for differences in original resolution between the different predictors. All predictors were then clipped to the study areas. We then calculated pairwise correlations for predictor variables based on 10,000 random points sampled from the study area. A rejection threshold (Spearman's |rS| > 0.7) was used to identify highly correlated predictors (Dormann et al. 2013). For each of the pairs exceeding this value, the predictor considered biologically least important for the Wallcreeper was removed based on literature and expert knowledge (Methods S5).

**TABLE 1:** Predictor variables used for the Wallcreeper species distribution models (italics: variable retained), unit of measurement, resolution, species‐specific ecological relevance, model in which the variable was retained (O: Wintering; B: Breeding; OB: Both wintering and breeding models) and data source.

Category	Predictor	Unit	Original resolution	Possible relationship	Model	Source
Topography	Elevation	m	25 × 25 m	Elevation optimum (Knaus et al. 2018)	None	DHM25 (1)
	*Sine of the aspect (eastness)*	−1 to 1	25 × 25 m	Importance of sun exposition (Luisier et al. 2022)	B	DHM25
	*Cosine of the aspect (northness)*	−1 to 1	25 × 25 m	Importance of sun exposition (Luisier et al. 2022)	B	DHM25
	*Standard deviation of the eastness (variability of exposition)*	0 to 1	25 × 25 m	Variability of exposition (Saniga 2004; Luisier et al. 2020)	OB	DHM25
	*Slope*	°C	25 × 25 m	Steep rock face	B	DHM25
Climate	*Average seasonal ambient temperature*	°C	100 × 100 m	Prey availability, thermal budget	OB	WSL (2)
	*Average seasonal precipitation*	mm	100 × 100 m	Foraging possibilities	OB	WSL
	*Average seasonal solar radiation*	WH/m^2^	25 × 25 m	Importance of sun exposition (Luisier et al. 2022)	OB	DHM25
	*Three biogeographical regions*	1 to 3	Vector	Effect of region	O	Gonseth and Sartori (2022)
Geology	*Limestone frequency*	%	Vector	Rock type importance (Saniga 2004)	B	gk200 (3)
	*Gneiss and granite frequency*	%	Vector	Rock type importance (Saniga 2004)	OB	gk200
	*Other rock frequency*	%	Vector	Rock type importance (Saniga 2004)	B	gk200
Land cover	*Scree frequency*	%	Vector	Foraging habitat availability (Luisier et al. 2020)	OB	Vector 25 (4)
	*Percent rock within 25 m cell*	%	Vector	Foraging habitat availability (Luisier et al. 2020)	OB	Vector 25
	Percent forest within 25 m cell	%	Vector	Indirect effect	None	Vector 25
	Percent shrubs within 25 m cell	%	Vector	Indirect effect	None	Vector 25
	Percent cultivation within 25 m cell	%	Vector	Indirect effect	None	Vector 25
	*Percent glacier within 25 m cell*	%	Vector	Indirect effect	O	Vector 25
	*Percent anthropogenic within 25m* ^a^	%	Vector	Indirect effect and habitat (Maumary et al. 2007)	OB	Vector 25
	Percent grassland and unproductive vegetation within 25 m cell	%	Vector	Indirect effect	None	Vector 25
	*Percent rock in a radius of 1 km*	%	Vector	Habitat availability and attractivity	OB	Vector 25
	*Percent gorge within 25 m cell*	%	Vector	Additional habitat availability (Saniga 2004)	B	Vector 25
Rock face characteristics	*Rock face planar area*	m^2^	Vector	Habitat availability and attractivity	O	swissTLM3D (5)
	*Distance to the next rock face*	m	Vector	Habitat availability and attractivity	OB	swissTLM3D
	Distance to the next grassland	m	Vector	Prey productivity	None	Grassland model (6)
	Distance to the next forest	m	Vector	Prey productivity	None	Vector 25
Connectivity	Connectivity between near rock faces	Degree	25 × 25m	Small scale connectivitiy	None	swissTLM3D and grainscape (7)
	Links to centroids	Amount	25 × 25m	Large scale connectivity	None	swissTLM3D and grainscape

*Note:* See Figures S3 and S4 for the permutation importance of each variable. (1) DHM25 is the digital height model of Switzerland (Swisstopo 2022): https://www.swisstopo.admin.ch/en/geodata/height/dhm25.html. (2) WSL is the Federal Institute for Forest, Snow and Landscape Research. (3) gk200 is a simplified geotechnical map of Switzerland 1:200,000 (Bundesamt für Statistik, 1967). (4) Vector 25 is a digital cartographic model of Switzerland 1:25,000: https://www.swisstopo.admin.ch/en/geodata/maps/smv/smv25.html. (5) swissTLM3D is the topographic model of Switzerland (Swisstopo 2022): https://www.swisstopo.admin.ch/en/geodata/maps/smv/smv25.html. (6) Grassland model is the model of permanent grassland in Switzerland developed by Pazúr et al. (2022). (7) *grainscape* package (Chubaty et al. 2020) see Methods S4.

^a^ Human infrastructures and settlements.

#### 2.2.4 Modeling

The analysis was performed in R 4.2.1 (R Development Core Team 2022) using RStudio 2022.7.1.554 (RStudio Team 2022). We modeled the distribution of the species using Maxent 3.4.3, a machine learning method based on the maximum entropy principle (Phillips et al. 2006). This approach is commonly used to model species distribution when only reliable presence data, but no absence data, are available and is robust for predicting future distributions under climate change (Brambilla et al. 2022). This method compares the values of the different predictors at the observation points with those of randomly drawn locations in the study area (i.e., background locations) (Phillips et al. 2006). For both wintering and breeding periods, we randomly split the field observations and background locations into two parts to create training (80%) and testing (20%) datasets. The training dataset was used in the next modeling steps to train the model, whereas the testing dataset was only used at the end of the process as an independent dataset to evaluate the final model performance. As Switzerland exhibits extreme landscape and topographical heterogeneity, we wanted the model to make meaningful predictions across the entire study area. We therefore fit a model for each period using spatial cross validation to evaluate model accuracy across the entire country. The study area was divided into eight latitudinal spatial blocks of the same width to produce four cross validation folders using the package *blockCV* (Valavi et al. 2019). We then used the function *spatialAutoRange* to perform an analysis of spatial autocorrelation among continuous variables, producing a minimum recommended side‐length (9727 m) for each block.

Training and evaluation of the model was done with the package *SDMtune* (Vignali et al. 2020), and we used the function *optimizeModel* to optimize model performance. This function uses a genetic algorithm to optimize the value of the hyperparameters (i.e., the parameters of the machine learning algorithm). We then used the *reduceVar* function to reduce the complexity of the model by excluding the predictor with less than 1% of permutation importance, except if the exclusion of the predictor reduced the Area Under the Curve (AUC) value (determined using a Jackknife procedure). Finally, we again used the *optimizeModel* function to optimize the performance of the new model. The variables could then be ranked by their permutation importance, which was obtained by measuring the decrease in AUC after having randomly permuted the environmental predictors' values within their range. Model performance was assessed using AUC.

#### 2.2.5 Climate Change Scenarios

Following the method of Barras, Braunisch, and Arlettaz (2021) and Barras, Niffenegger, et al. (2021), we projected the distribution of the species into the future using two climatic variables available in our study area: precipitation and temperature. They were available as rasters at 100 m resolution for the years 2030–2050 (short term predictions over approximately 15 years) and 2080–2100 (long term predictions over approximately 65 years) based on predictions from the European branch of the coordinated regional climate downscaling experiment, EURO‐CORDEX (www.euro‐cordex.net; 0.11‐degree resolution). For each of these time intervals, the CNRM‐CERFACS‐CNRM‐CM5 model with the moderate scenario RCP 4.5 (rcp45) and an extreme scenario RCP 8.5 (rcp85) was applied to project our models (for breeding and wintering period), resulting in four different future predictions per time interval. Rcp45 reflects a decrease in greenhouse gas emissions as early as 2050, whereas rcp85 reflects a continuous increase in emissions until 2100 (IPCC 2014). All data were provided on request by the Swiss Federal Institute for Forest, Snow and Landscape Research (WSL).

### 2.3 Estimation of Abundance

#### 2.3.1 Study Area and Sampling Design

The abundance of Wallcreeper is notoriously difficult to estimate due to (1) large territories spread over inaccessible alpine terrain and (2) extremely secretive behavior that limits detectability. For this reason, no reliable estimates exist for Wallcreeper abundance at the regional scale in Switzerland. Given such logistical constraints, we were unable to survey the species over the entire country and instead focused on the Alps in the cantons of Valais and Vaud to assess Wallcreeper abundance. This area covers 5941 km^2^, which represents only 4% of the total alpine area in Switzerland, but 19.7% of Swiss mountains. Our study area represents more than 10% of the core Wallcreeper range in Europe (Keller et al. 2020).

To create our field sampling design, we first divided the study area into 500 × 500 m squares (25 ha) by subdividing the 1 km^2^ squares used in long‐term bird monitoring and atlas programs by the Swiss Ornithological Institute (Knaus et al. 2018; Schmid et al. 2004). Second, to include the entire gradient of habitat suitability and rock face area in the sampled squares, we categorized the sample in eight strata using two stratifications. The first stratification was based on the occurrence probability of the country‐wide distribution models: < 0.25, 0.25–0.50, 0.50–0.75 and > 0.75. The second indicated whether each square included more or less than 50% cell classified as rock face in distribution models. We also discarded two types of squares: (1) squares with a mean altitude higher than 2200 m in the wintering period or 3200 m in the breeding period due to accessibility constraints and (2) squares with insufficient rock face area to maintain a Wallcreeper territory (fewer than thirty‐two 25 × 25 m cells considered as rock face in distribution models). Next, we randomly picked one square per stratum for the wintering period and two squares per stratum for the breeding period. The wintering survey was reduced in comparison to the breeding survey because the time available for sampling was shorter (less daylight in winter) and the species distribution is limited to a smaller spatial extent. Squares that were too dangerous (risk of avalanche or rock fall, etc.) or too close from one another to be independent with certitude (i.e., with a distance smaller than 4 km between wintering and 2 km between breeding sites) were replaced by another randomly sampled square from the same stratum. Finally, we spatially balanced the sampling to have the same number of squares per occurrence probability stratum in each part of the study area (east and west of the city of Sion, following natural borders) (Figure S2). Due to small amounts of rock face in two wintering squares, we added two squares in the respective strata, which produced a final sample of 18 and 32 squares, for the breeding and wintering periods, respectively (Table S1). The field survey protocol for each period was designed to maximize detection probabilities, taking into account period‐specific behavior and individual identification based on photographs (see Methods S2 for the detailed protocol).

#### 2.3.2 Abundance Modeling

To estimate Wallcreeper abundance, we used multinomial N‐mixture models, which account for imperfect detection and territory identity (Kéry and Royle 2016; Royle 2004). We fit models in R using the *multinomPois* function in the package *unmarked* (Fiske and Chandler 2011). As a single abundance covariate, we included the species' mean occurrence probability of the whole square predicted by the respective distribution model fit previously in Maxent. It was obtained by setting 25 × 25 m cells not touching the rock face to zero occurrence probability and then averaging over the whole square. For both periods, we used covariates potentially affecting detection probability which included the visit number (1, 2, or 3), the proportion of rock face exposed to the sun during a visit (0 to 1) and the mean ambient temperature during the visit in °C. For the breeding model, we also included the time at which the visit started after dawn in hours and the fledging date of the chicks in each square, which was estimated either by direct observation or deduction from the adult behaviour (Luisier et al. 2020). If no Wallcreeper was detected in the square, we estimated a date based on expert knowledge, knowing that the fledging date in normal conditions (around 1800–2400 m or in most gorge habitats) is around 15 July, but is earlier at lower altitudes and later at higher altitudes. We then fixed the starting date of the covariate 10 days after the estimated fledging date to include possible contacts concerning family with dependent chicks.

Using all combinations of detection covariates, the obtained models were ranked using Akaike's Information Criterion (AIC) (Kéry 2018). We assessed the goodness of fit using two basic metrics, the root‐mean‐square error and the mean absolute error, and we performed a visual inspection by comparing observed and fitted values. As recommended by Kéry and Royle (2016), we also performed a parametric bootstrap (1000 simulations) to assess the fit with the c‐hat using the function *Nmix.gof.test* and three fit statistics: Chi‐square (equivalent to the *Nmix.gof.test* without the c‐hat), the sums‐of‐squares and the Freeman‐Tukey statistic. According to these metrics, we selected the best fitting model of the top ranked models, including at most three additive covariates in order to maintain parsimony as all top‐ranked models gave similar estimates (Kéry and Royle 2016).

For the abundance projection, we calculated the mean occurrence probability of each 500 × 500 m squares of each distribution projection. We then used these to project the abundance models to the Valais/Vaud study area for the current and the four future scenarios and both seasons. To account for the fact that the area that was effectively sampled was larger than the area in which we carried out the counts and therefore could result in population size overestimation, we divided the predicted abundance in each square by 1.48. This value was obtained by following the method of Kéry and Royle (2016), which assumes territories as being circular and allows to keep only the part of the circle that is in the square, and based on an average wintering territory planar area of 2.5 ha in good quality sites (mean occurrence probability > 0.5) and 5 ha in poor quality sites (mean occurrence probability ≤ 0.5). This estimate of territory size was obtained from counts carried out during the wintering period on 11 large rock face complexes in the same study area the two previous years (Luisier et al. 2022).

Each projection was then aggregated to 1 km^2^ resolution by summing the values of four touching 500 × 500 m squares with a predicted abundance higher than 0.25. This permitted us to better match the species biology in considering territories being between up to four low predicted abundance squares, whereas they would be discarded otherwise, and to match with the abundance spatial resolution for other species in Switzerland (e.g., Knaus et al. 2018). We then considered the 1 km‐squares with a predicted abundance lower than 0.75 as absences and summed up the values of all the other squares to get the total predicted abundance in the study area for each projection. Ninety‐five percent confidence intervals were obtained using a non‐parametric bootstrap procedure with 100,000 repetitions. For each iteration, a dataset was resampled (with replacement) from the original data, the N‐mixture model was refitted, and total abundance was re‐estimated after the same spatial procedure described above. Implausible values (< 100 or > 10,000) were discarded and 95% confidence intervals were calculated as the 2.5% and 97.5% of the bootstrapped distribution.

### 2.4 Analysis of the Predictions

The occurrence probabilities of the future projections were subtracted from the current projections to visually determine the areas with the greatest change in habitat suitability. We also calculated the maximum training sensitivity plus specificity *Cloglog* threshold of the model to convert the occurrence probability maps into presence/absence maps (Liu et al. 2013) to calculate the change in range size.

To have an altitudinal overview of the species distribution, we divided the study area in altitudinal bands of 100 m (e.g., from 400 to 499, 500 to 599 m, etc.). From the presence/absence maps, we then extracted only the cells on, or intersecting the rock faces to restrict the rest of the analysis to the habitat of the species (i.e., only including rock faces without the buffer area). The extracted cells could be then easily converted to area by multiplying them by their surface (= 625 m^2^). We first determined the mean altitude of presence cells. We then divided the area of predicted presence in each band by the total study area to obtain the proportion of predicted suitable area that represents each band. We did the same as for the difference maps to get the difference for each band between the current and the future projections. Finally, inside of each band, we divided the presence area by the total area of rock faces of the band to have the proportion of the band predicted as suitable. For the abundance projections, we did a similar altitudinal analysis with the predicted number of Wallcreeper pairs in each altitudinal band instead of the area.

## 3 Results

### 3.1 Models Performance and Thresholds

#### 3.1.1 Distribution Models

The *Cloglog* thresholds to convert occurrence probability into presence or absence were determined as 0.148 for the wintering model and 0.328 for the breeding model. The final models showed good to excellent performance: the training and testing AUC were respectively 0.928 and 0.914 for the wintering model, and 0.888 and 0.882 for the breeding model. Intensive field observations during abundance sampling also validated the results of the distribution models for the Alps in the Valais and Vaud study area, with 92% (wintering) and 94% (breeding) of the field observations made within predicted presence cells with predicted occurrence.

#### 3.1.2 Abundance Models

All top‐ranking AIC models gave similar abundance estimates but variable detection probability estimates. For the wintering and the breeding periods, the selected models showed a good fit (Tables S2–S4).

### 3.2 Distribution Models: Predictor Importance

#### 3.2.1 Wintering Model

**FIGURE 2:**
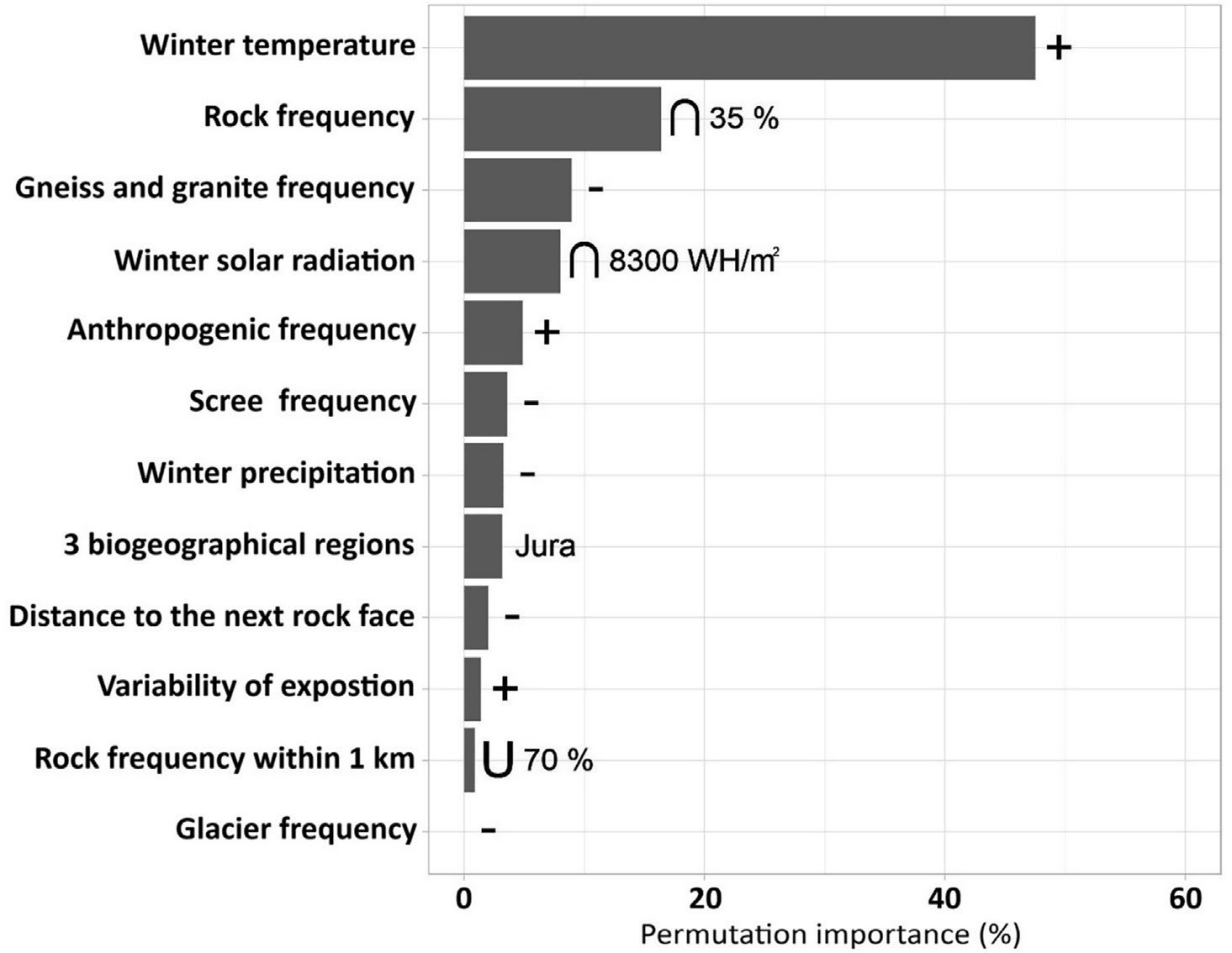
Permutation importance of the environmental predictors used in the Maxent model for predicting the probability of occurrence of wintering Wallcreepers in Switzerland. The symbol next to the bars depicts the response curve type of a univariate model trained using one variable at a time, with + indicating a positive response type, − negative, ∩ unimodal with one optimum and U unimodal with one minimum. Optimum and minimum values are indicated after the curve type. For the categorical variable, the best category is indicated. The permutation importance was obtained by randomly permuting the values of the respective predictor within its range and is represented as the decrease in the training AUC (%) normalized to percentage. See Table 1 for predictor explanation.

Twelve predictors were retained (Figure 2). The most important predictor explaining the species occurrence was the average winter ambient temperature (i.e., December to February) with a permutation importance (PI) of 47.5% and occurrence probability increasing with warmer temperatures. The second most important predictor was the rock availability (percentage within a 25 m raster cell) (PI = 16.4%) with an optimum at 35%. Winter precipitation, the other climate predictor used in future projections, was ranked 7th (PI = 3.3%), with preference for low precipitation levels.

#### 3.2.2 Breeding Model

**FIGURE 3:**
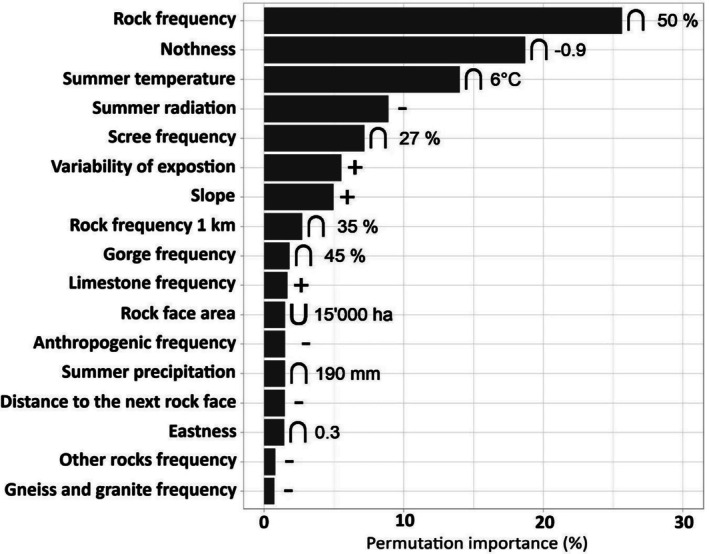
Permutation importance of the environmental predictors used in the Maxent model for predicting the probability of occurrence of breeding Wallcreepers in Switzerland. The symbol next to the bars depicts the response curve type of a univariate model trained using one variable at a time, with + indicating a positive response type, − negative, ∩ unimodal with one optimum and U unimodal with one minimum. Optimum and minimum values are indicated after the curve type. The permutation importance was obtained by randomly permuting the values of the respective predictor within its range and is represented as the decrease in the training AUC (%) normalized to percentage. See Table 1 for predictor explanation.

Seventeen predictors were retained (Figure 3). Wallcreepers were associated with rock availability (percentage within a 25 m cell) between 25% and 75% (PI = 25.6%) and were less likely to occur in strict southern and broadly northern expositions (northness predictor; PI = 18.7%). Among the remaining climatic predictors used in future projections, occurrence probability increased at moderate breeding ambient temperatures (i.e., May to July), with a clear optimum around 6°C (ranked 3rd, PI = 14%) and medium breeding precipitation (ranked 13th, PI = 1.5%), with an optimum at 190 mm over these 3 months.

### 3.3 Model Predictions

#### 3.3.1 Current Predictions

**FIGURE 4:**
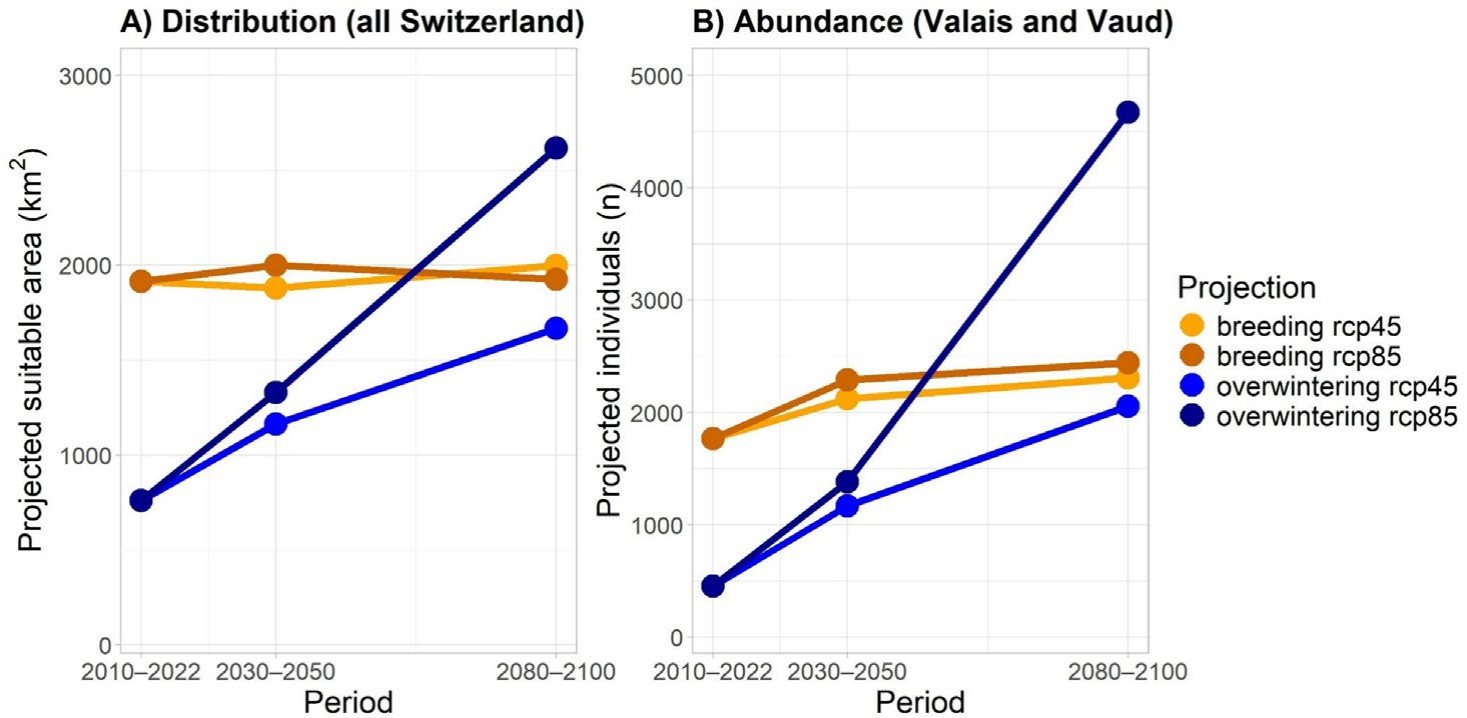
Predictions of Wallcreeper distribution (A) and abundance (B) for breeding and wintering periods in Switzerland (distribution) and its southwestern part (abundance) under current conditions (2010–2022) and for two future time intervals (2030–2050 and 2080–2100) given two projected climate change scenarios (rcp45 and rcp85 are for moderate and extreme greenhouse gas emission scenarios, respectively). The number of individuals for the breeding period was obtained by multiplying the predicted number of pairs by two (see Methods). Exact figures are in Tables S5 and S6.

A total of 761 km^2^ and 1915 km^2^ were predicted to be currently suitable for the species for the wintering and breeding periods, respectively (Figure 4). This represented respectively 14.4% and 38.9% of the planar area of rock faces found in the period‐specific study areas (Tables S5 and S6). The predicted suitable area (i.e., presence cells) overlapping between the two periods was 517.7 km^2^ (68% and 27% of the wintering and breeding predicted suitable area, respectively) (Figure 8). The abundance models restricted to Valais and Vaud predicted an overwintering population of 454 individuals (predicted 95% CI: 125–2370) and a breeding population of 884 pairs (predicted 95% CI: 306–3103) (Figure 4).

**FIGURE 5:**
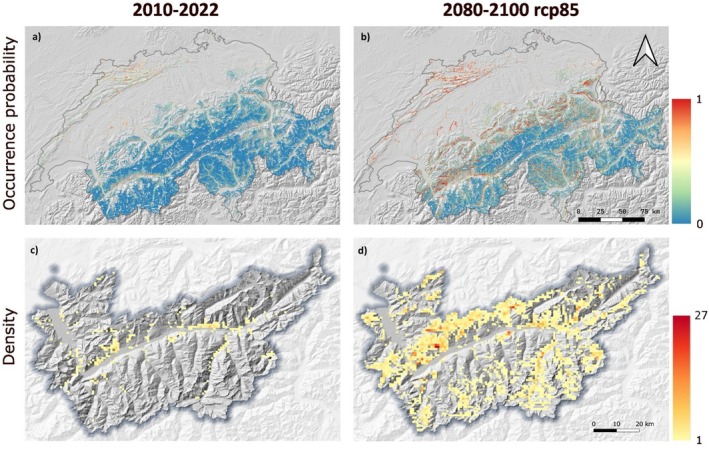
(a, b) Predicted occurrence probabilities of wintering Wallcreepers in Switzerland for the periods 2010–2022 and 2080–2100 under the climate change scenario rcp85. (c, d) Population density (number of predicted overwintering territories per km^2^) in the Valais and Vaud study area for 2010–2022 and 2080–2100 under rcp85.

During the wintering period, the highest occurrence probabilities and the most suitable altitudinal bands were predicted to be below 1000 m a.s.l. (Figure 5). Most of the suitable area was predicted to be in the Alps (Figure S3), with a mean altitude of 1383 m a.s.l. compared with a mean altitude of rock face of 2221 m a.s.l. Eighty‐two percent of the total predicted suitable area was in altitudinal bands representing each more than 4% of the total suitable area (Figure S4). All of these bands were between 600 and 2100 m a.s.l. The abundance model restricted to the Alps in Valais and Vaud showed similar patterns with a mean altitude of the territories of 1253 m a.s.l. (Figure 5).

**FIGURE 6:**
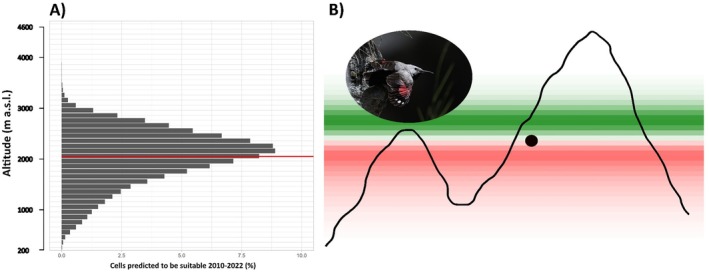
Breeding altitudinal distributions of the Wallcreeper in Switzerland based on the country‐wide species distribution model for (A) current conditions (2010–2022) and (B) the projected climate scenario rcp85 for 2080–2100. For both panels, the y‐axis represents 100 m altitudinal bands. (A) Percentage of cells predicted to be presently suitable (red horizonal line is the mean). Bars sum to 100%, representing the entire predicted suitable area. (B) Difference in predicted suitable breeding area between current conditions and the 2080–2100 rcp85 climate scenario. Green represents elevational gains and red losses, with the color intensity indicating the relative magnitude of the difference per elevational band. The black dot represents the average elevation of the predicted suitable area in 2080–2100 under rcp85. The black curves conceptually depict mountain ranges of different altitudes, visualizing likely declines in distribution and population size across medium elevation massifs common to the species' range across Europe and likely increases across the high massifs of the Swiss Alps.

**FIGURE 7:**
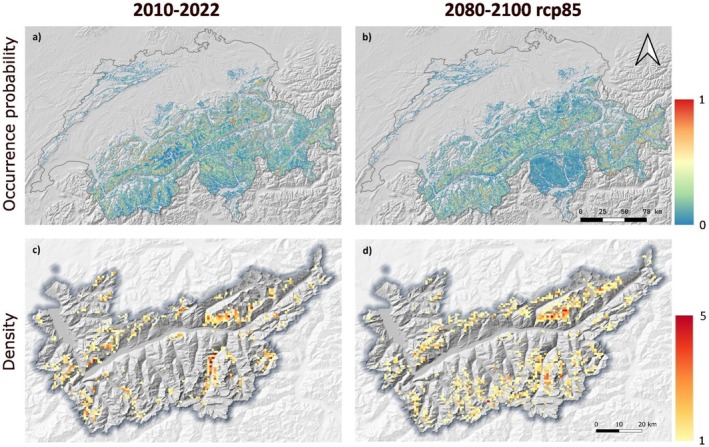
(a, b) Predicted breeding occurrence probabilities of Wallcreepers in Switzerland for the time intervals 2010–2022 and 2080–2100 under the climate change scenario rcp85. (c, d) Predicted wintering density (number of wintering territories predicted per km^2^) in the Valais and Vaud study area for 2010–2022 and 2080–2100 under rcp85.

During the breeding period, the most suitable altitudinal bands were predicted to be between 1400 and 2300 m a.s.l. (Figure 7). 99.7% of the predicted suitable area was in the Alps (Figure S5), with a mean altitude of 2038 m a.s.l., compared with a mean altitude of rock face of 2225 m a.s.l. (Figure 6). Seventy‐three percent of the total predicted suitable area was in altitudinal bands representing each more than 4% of the total suitable area. All of these bands were between 1600 and 2700 m a.s.l. The abundance model predicted similar patterns with a mean altitude of the territories 2119 m a.s.l. and bands with 100 pairs or more predicted between 1800 and 2600 m a.s.l.

#### 3.3.2 Future Predictions

All future wintering projections predicted an increase of the suitable area across Switzerland and abundance in the Alps in Valais and Vaud, with the time interval 2030–2050 being highly similar for both scenarios (rcp45 and rcp85) and predicting an increase of up to 1164 km^2^ (982 individuals) and 1331 km^2^ (1179 individuals), respectively. Predictions for the long‐term scenario differed more, with respective totals of 1667 km^2^ (1776 individuals) and 2616 km^2^ (4095 ind.) (Figure 4; Figure S3).

For the breeding period, increases in occurrence probability, suitable area, and altitudinal band suitability were generally predicted above 2300–2400 m a.s.l. in the Alps for all periods and scenarios, whereas below this altitude, the opposite trend was predicted (Figure 6). The overall suitable area was not predicted to change by more than 4.4% in comparison to the current one (Figure 4). The three biogeographical regions with a mean altitude of rock faces below 2400 m were predicted to exhibit decreases in suitable area, between −16.1% and −57.8% for the 2080–2100 rcp85 scenario. The two other regions, with the mean altitude above 2400 m, were predicted to follow the opposite trend with an increase up to +40.8% in the Eastern central Alps (Figure 7; Figure S5).

**FIGURE 8:**
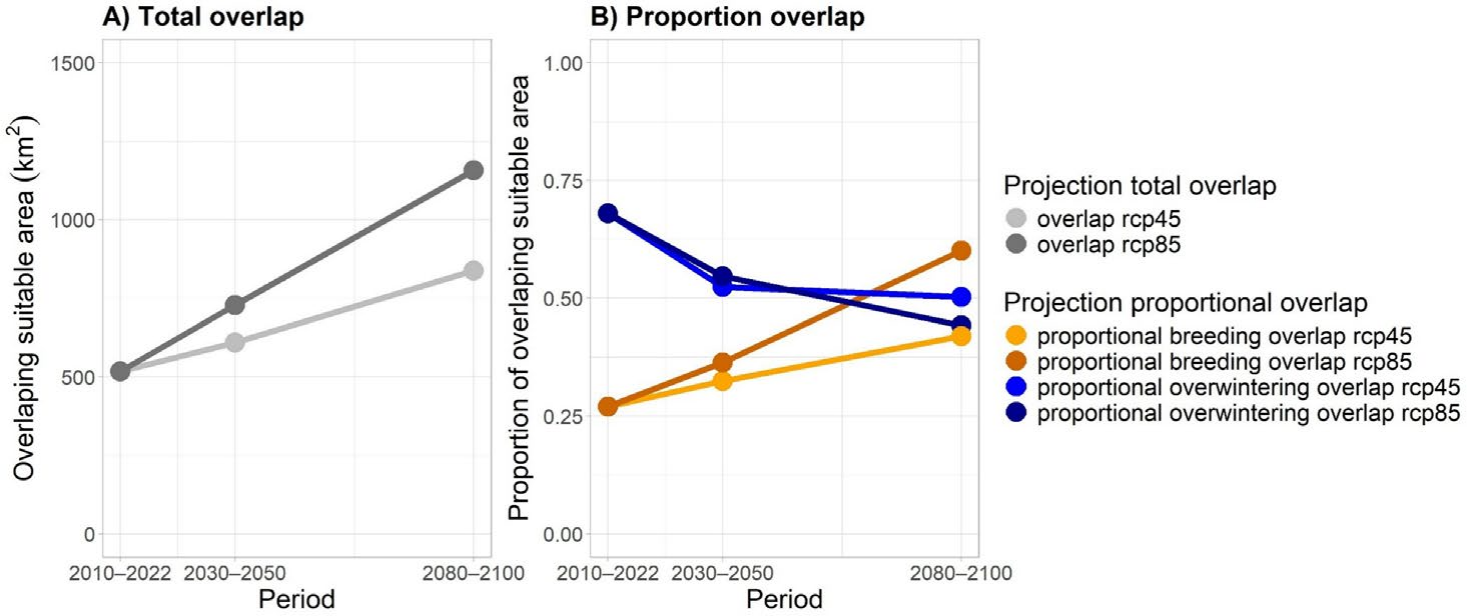
(A) Absolute changes in distributional overlap of the breeding and nonbreeding ranges for the Wallcreeper in Switzerland for current conditions (2010–2022) and under two projected climate change scenarios (rcp45 and rcp85). (B) Proportional change in distributional overlap by period and climate scenario.

The abundance predictions showed a similar pattern but changed more with the severity of the scenario (Figure 4). The predicted suitable area overlapping between the two periods was predicted to increase to 1157 km^2^ (+123%), with the proportion of wintering area in the overlap decreasing from the current 68% down to 44% and the proportion of breeding area in the overlap increasing from the current 27% to 60% (Figure 8).

## 4 Discussion

Including both distribution and abundance predictions, we present a novel annual life‐cycle analysis at a high spatial resolution for the possible effects of climate change on a high elevation species using SDMs. Overall, we revealed contrasting seasonal predictions. During the breeding season, our model predicted an upslope elevation shift with little change to overall area, whereas the wintering range was predicted to expand up to 244%. This led to an increase in overlap between breeding and wintering ranges by 123%. Using two different climate scenarios across two different time projections, our modeling approach predicted (1) overall range expansions for the species across its distribution in Switzerland, (2) severe breeding area contractions at its lower elevational limits, and (3) future colonization of high elevation areas during the wintering period. Our abundance models based on intensive field surveys showed similar results with increased precision and biological realism. Overall, we demonstrate how predictive models can accurately embrace complex life history strategies and emphasize the central role that the Swiss Alps will play in conserving the species in Europe.

### 4.1 Distribution Model Outputs and Species Ecology

The good to excellent AUC of the models and the validation of the predicted presence by the field observations of abundance indicates that our models can predict with sufficient accuracy the current distribution of the species (Guisan et al. 2017). Furthermore, our wintering model highlighted that the distribution of Wallcreepers within the study area was largely related to the average winter ambient temperature (PI = 47.5%), emphasizing the potential sensitivity of the species to climate change. While climate is often a weaker predictor of avian occurrence than habitat (Brambilla et al. 2019), the opposite is often exhibited for high elevation montane species, which have specialized thermal niches (e.g., Resano‐Mayor et al. 2019; Barras, Braunisch, and Arlettaz 2021; Barras, Niffenegger, et al. 2021; de Gabriel Hernando et al. 2021). This is particularly relevant for the Wallcreeper given its reliance on exposed rock faces that are not expected to undergo significant changes in habitat structure or composition. Conversely, climatic predictors had a lower importance for the breeding period, with the breeding temperature being ranked 3rd (PI = 14%). As the optimal breeding temperature was 6°C and the response to the wintering temperature was positive with optimal areas at 3.4°C, the ideal temperatures for wintering are probably not currently available in the study area but may be attained in the future. Hence, ambient temperature is currently likely to be a much more limiting factor during the wintering period than during the breeding season. An additional explanation could be that the restricted extent of the breeding study area only encompassed mountainous regions where ambient temperature is closer to the species' optimum than in the excluded lower elevation regions (i.e., the Swiss Plateau).

The strong association with higher temperatures during the wintering period likely reflects two underlying processes. First, the Wallcreeper's main prey, rock‐crevice arthropods, are more likely to become active under warmer winter conditions, which enhances their availability (i.e., abundance modulated by accessibility) (Høye and Forchhammer 2008; Luisier et al. 2022). Second, Wallcreepers likely save energy by avoiding increased metabolic output at extremely cold temperatures (Johnson 1968). The two other climatic factors retained in the models, solar radiation and precipitation, should have similar effects given that low precipitation and high solar radiation in suboptimal thermal environments also favor arthropod activity while enhancing bird thermoregulation (Høye and Forchhammer 2008; Peng et al. 1992; Waringer 1991). On the other hand, cliffs are prone to comparatively rapid overheating, mainly due to interactions between ambient air temperature, the angle of solar radiation, and the degree to which different geological substrates absorb or radiate heat (Hasler et al. 2011; Molaro and Mckay 2010). Combined, these factors could establish suitable thermal environments in winter, while exceeding physiological thermal limits in the summer (Johnson 1968). This may explain why breeding Wallcreepers avoid sun‐exposed rock configurations, nesting instead at shady places or in deep holes (Luisier et al. 2020; Saniga 2004). Similarly, overly hot sites may also hamper arthropod development, that is, negatively influence food availability (Luisier et al. 2020; Saniga 2004). Our models thus support field observations that Wallcreepers seek optimal microclimates in rock faces, in conformity with their physiological and trophic requirements (Briscoe et al. 2023).

### 4.2 General Predictions

Our study makes fundamental advancements in our knowledge of Wallcreeper distributions and population size in central Europe. Given that the species is an exclusive inhabitant of rock faces that are often difficult to survey due to accessibility issues, our models make it possible to predict potentially suitable areas without requiring logistically complex surveys. Similarly, this study provides the first reliable estimates of the Wallcreeper's population size in Switzerland, mainly due to the challenges of effectively monitoring such an unusually cryptic and inaccessible species. The low number of 387 wintering individuals is explained by the limited number of suitable cliff systems at low elevation (Maumary et al. 2007). Our estimates of 1189 pairs for the southwestern part of the Swiss breeding population alone (encompassing 19.7% of mountains in Switzerland) are proportionally much higher than the former country‐wide estimates of 1000–2500 pairs in 2013–2016 (Knaus et al. 2018). Switzerland may well harbor a much larger population size than previously estimated at around 6000 breeding pairs. While we recognize that the magnitude of the confidence intervals for population size estimates is wide (mostly due to small sample sizes), all models yielded very similar point estimates.

### 4.3 Range Expansion and Overlap

By leveraging such advancements in modeling and intensive survey efforts, we predicted generally positive trends in the probability of occurrence, distribution extent and abundance of Wallcreepers toward higher elevations, especially in winter. The northern portion of the Alps appears to be particularly important for future range expansions, emphasizing how topographies associated with cooler microclimates will play a central role for alpine diversity. This contrasts with the pessimistic predictions concerning other high elevation specialists in western and central Europe, which mostly project contractions of their breeding ranges by the middle of the century (Brambilla et al. 2022; Scridel et al. 2018). These discrepancies may have arisen because (1) we also considered the wintering period while most studies focus on the breeding period only, and (2) Switzerland harbors most of the highest mountains of Europe, thus providing room for upslope movements for range adjustments (a shift that is out of scope in low elevation mountain massifs).

The projected expansion of the wintering area is predicted to contribute to an increase in the overlap in space use between the wintering and breeding periods. Such expansion of the wintering area due to climate change, rather than a shift, has already been described for species such as shorebirds in England (Maclean et al. 2008). However, these changes in overlap have rarely been studied, and mainly in a latitudinal context (e.g., Ambrosini et al. 2016). A typical example is the European Robin ( *Erithacus rubecula* ), in which southern breeding populations are largely sedentary, while northern populations are strictly migratory, with wintering mostly occurring in the breeding range of the southern populations (Fandos and Tellería 2020; Korner‐Nievergelt et al. 2014). It is currently unknown if a similar phenomenon exists for altitudinal migrants (e.g., if birds breeding higher up migrate more than the one at lowest elevations).

### 4.4 Regional Trends Within the European Context

Our models predict a marked expansion of wintering range but no change in the overall breeding area of the Wallcreeper within Switzerland. However, a dramatic upward altitudinal shift in distribution is projected, with a massive area loss (−58%) below 2300–2400 m a.sl. Given that much of the European population breeds at these lower elevations, the Wallcreeper may be dramatically affected in the future across the continent. As an example, the models suggest that the relatively low Jura mountains may fail to maintain a viable breeding population in the short term. Past coarse‐grained (1 km^2^ resolution) distribution modeling for the species in southwestern Europe predicted a decrease in suitable area of −6% to −7% and −27% to −35%, during breeding and wintering, respectively, by 2041–2060 (de Gabriel Hernando et al. 2021). However, it is likely that the limited loss in the breeding range results from an under‐estimation of lost space merely due to low resolution modeling.

The Wallcreeper seems to follow the global breeding trend predicted for typical high elevation specialists, with strong losses in suitable areas at the lower elevations and stability to expansion higher up (Brambilla et al. 2022; Scridel et al. 2017, 2018). Yet, unlike most high‐elevation specialists, we believe it will probably maintain relictual populations at lower elevations due to its very peculiar species‐specific habitat requirements, notably its obligate reliance on rock faces. In effect, its habitat will undergo only limited structural changes in comparison with the mostly vegetated—even if only partly—habitat of other high elevation specialists (Malfasi and Cannone 2020). In particular, cooler breeding sites such as large gorges situated at low elevation may offer some buffering against the adverse effects of climate warming (Alessandrini et al. 2022; Luisier et al. 2020), as already documented in the West Carpathians (Saniga 1995, 2004). In general, however, countries with high mountains like Switzerland will have an increasing responsibility for the conservation of the species (Keller and Bollmann 2001).

### 4.5 Study Limitations and Perspectives

Like most predictive studies, our results consider habitat suitability but no other factors that operate in a metapopulation system, such as habitat connectivity, prey supply and population dynamics (Howard et al. 2023). Using a rock face obligate species as a research model largely avoids the common issue of predicting concurrent changes in land cover, excepting rocky areas that will be exposed by glacier retreat. However, between 1990 and 2015, approximately 7.8 km^2^ of rocky terrain emerged after glacial melting in the Swiss Alps, representing only 1% of the current predicted suitable area for the Wallcreeper (Zekollari et al. 2019). That said, an additional 908.5 km^2^ is predicted to be free form ice by 2080–2100, which may reveal many potential new habitats. Other environmental descriptors were not considered in this study due to a lack of extant databases. The complexity of the cliff microhabitat structure (cracks, holes, overhangs, fine‐grained aspect variation) could not be included in our 2D models, despite playing an important role for occupancy (Luisier et al. 2020; Saniga 2004). Projected temporal and altitudinal changes in ecological interactions (arthropod vs. vegetation communities, which determines food supply for insectivorous species) could also not be accounted for in our models although they will likely play a major role in shaping future ecosystem functions (Pellissier et al. 2018). Likewise, observations on human constructions (buildings, bridges) in winter, though minimal, were not accounted for in our models. Finally, population monitoring in a fraction of the study area provides an independent expert‐based assessment that our current population projections are indeed realistic (Luisier 2022; Luisier et al. 2022).

### 4.6 Conservation Implications

Our models stress again the key role played by the Swiss Alps for the conservation of montane biodiversity in Europe (Brambilla et al. 2022; Keller and Bollmann 2001; Vittoz et al. 2013). They furthermore allow the precise identification of future suitable Wallcreeper areas in Switzerland, offering a sound base for spatially prioritizing conservation action. Following Brambilla et al. (2022), we recommend that the highest quality areas, which will likely serve as climatic refugia for the species, receive nature conservation status if not yet embedded within reserves. This would be a decisive step given that, among the four main countries in the Alps, Switzerland is the one with the smallest fraction of its territory considered as climate refugia (18%) under a form of nature protection status (Brambilla et al. 2022). In rock faces inhabited by Wallcreepers, measures to manage human leisure activities are recommended. For instance, rock climbing activities, which are rapidly spreading, should avoid the removal of valuable vegetated microhabitats and their arthropod communities that form the bulk of Wallcreeper's diet (Camp and Knight 1998; Clark and Hessl 2015). Finally, given its high habitat quality requirements and its charisma and beauty, and the fact that rock face conservation will benefit other high‐elevation and rock face species (Maggini et al. 2014), the Wallcreeper might in the future be elected in its dual role of an umbrella and flagship species within the montane rock face community for raising public awareness and enhancing conservation support (March‐Salas et al. 2023).

## 5 Conclusions

Using different modeling approaches that include citizen‐science presence data and novel field‐collected abundance data, we predicted an upward altitudinal shift of the Wallcreeper breeding range while its overall extent and population size are expected to remain relatively stable. In winter, suitable habitat is predicted to expand toward higher elevations. These results suggest that climate change may have positive implications for the species' range, predictions that are rarely reported for cold‐oriented species. Because the Wallcreeper only lives on rock faces, our predictions are likely immune to usual biases in SDM studies, such as changes to land cover that are difficult to predict. As one of the most specialized cliff‐dwelling birds, our models can be used to understand how other mountain breeding in rock faces would respond to future climate change. Future research should quantify the extent to which these patterns could apply to other cliff‐nesting mountain birds and investigate the underlying mechanisms, particularly the role of food resources and nesting‐site availability. Furthermore, basic knowledge about seasonal movement of alpine birds, especially the Wallcreeper, remains scarce and is essential for better understanding potential implications of global change. More broadly, our findings highlight the importance of the Swiss Alps as a climate refuge for alpine biodiversity and emphasize the need to protect habitats that may support viable populations under future climatic conditions.

## Author Contributions

**Célestin Luisier:** conceptualization (lead), data curation (lead), formal analysis (lead), funding acquisition (equal), investigation (lead), methodology (lead), project administration (lead), visualization (lead), writing – original draft (lead), writing – review and editing (equal). **Sergio Vignali:** conceptualization (supporting), data curation (supporting), formal analysis (equal), investigation (supporting), methodology (equal), software (lead), supervision (equal), validation (lead), visualization (equal), writing – review and editing (equal). **Veronika Braunisch:** formal analysis (supporting), investigation (supporting), methodology (supporting), writing – review and editing (supporting). **Arnaud G. Barras:** investigation (supporting), writing – review and editing (supporting). **Marc Kéry:** formal analysis (supporting), methodology (supporting), supervision (supporting), validation (supporting), writing – review and editing (supporting). **Raphaël Arlettaz:** conceptualization (equal), funding acquisition (lead), investigation (equal), project administration (equal), resources (equal), supervision (equal), writing – review and editing (equal). **Ian J. Ausprey:** conceptualization (equal), investigation (supporting), methodology (supporting), project administration (supporting), supervision (equal), visualization (supporting), writing – review and editing (equal).

## Funding

This work was supported by the Ignace Mariétan Foundation and Nos Oiseaux.

## Conflicts of Interest

The authors declare no conflicts of interest.

## Supporting information


**Figure S1:** Flowchart presenting the selection process of Wallcreeper presence data and number of locations retained after each filtering step for the breeding (red) and the overwintering (blue) periods.
**Figure S2:** Abundance sampling sites for the overwintering (blue) and breeding (red) periods in the abundance study area and location of the study area in black within Switzerland (top left).
**Figure S3:** Predicted area of Wallcreeper winter distribution for the six biogeographical regions of Switzerland included in the overwintering distribution model in alphabetic order.
**Figure S4:** Percent difference in Wallcreeper overwintering area (i.e., presence cells) between the current four future predictions (2030–2050) under rcp45 (A) and rcp85 (B) and 2080–2100 under rcp45 (C) and rcp85 (D), calculated for altitude bands of 100 m based on the species distribution model.
**Figure S5:** Predicted area of Wallcreeper distribution for the five biogeographical regions of Switzerland (see Figure S6) included in the breeding distribution model in alphabetic order.
**Figure S6:** Map of the biogeographic regions of Switzerland.
**Table S1:** Strata for selecting the sampling locations for the abundance models with the number of 500 × 500 m squares (25 ha) randomly sampled in each stratum.
**Table S2:** Parameter estimates, with standard error (SE) and 95% confidence intervals (CI) of the multinomial N‐mixture model used to predict Wallcreeper abundance for the overwintering period.
**Table S3:** Parameter estimates, with standard error (SE) and 95% confidence intervals (CI), of the N‐mixture model used to predict abundance for the breeding period.
**Table S4:** The three different metrics to assess model goodness of fit of multinomial N‐mixture models provided in the unmarked package, calculated using a parametric bootstrap with the function parboot separately for winter and the breeding modelsSee Kéry and Royle (2016) for details.
**Table S5:** Table for the winter period showing a comparison between the current distribution prediction 2010–2022 and the four different future distribution predictions (i.e., short term time interval 2030–2050 and long term time interval 2080–2100 each under moderate greenhouse gas emission rcp45 scenario and extreme greenhouse gas emission scenario rcp85) with the number of suitable cells, the projected suitable area (km^2^), the percentage of the total area and the percentage of difference since 2010–2022.
**Table S6:** Comparison between the prediction of the current breeding distribution 2010–2022 and the four different future breeding distribution predictions (i.e., short term time interval 2030–2050 and long term time interval 2080–2100 each under moderate greenhouse gas emission rcp45 scenario and extreme greenhouse gas emission scenario rcp85), with the number of suitable cells, the projected suitable area (km^2^), the percentage of the total area and the percentage of variation since 2010–2022.

## Data Availability

All data and code are available at: https://doi.org/10.6084/m9.figshare.31463131.
