# Supplementary material for: Climate Change Predicted to Trigger an Upward Altitudinal Range Shift and Boost the Abundance of a Montane Rock Face Specialist, the Wallcreeper (Tichodroma muraria)
Source: Ecol Evol. 2026 Jul 19;16(7):e73963. doi: 10.1002/ece3.73963 (PMC13381094; doi:10.1002/ece3.73963)
Supplement: Supplementary file 1 — Figure S1: Flowchart presenting the selection process of Wallcreeper presence data and number of locations retained after each filtering step for the breeding (red) and the overwintering (blue) periods. Figure S2: Abundance sampling sites for the overwintering (blue) and breeding (red) periods in the abundance study area and location of the study area in black within Switzerland (top left). Figure S3: Predicted area of Wallcreeper winter distribution for the six biogeographical regions of Switzerland included in the overwintering distribution model in alphabetic order. Figure S4: Percent difference in Wallcreeper overwintering area (i.e., presence cells) between the current four future predictions (2030–2050) under rcp45 (A) and rcp85 (B) and 2080–2100 under rcp45 (C) and rcp85 (D), calculated for altitude bands of 100 m based on the species distribution model. Figure S5: Predicted area of Wallcreeper distribution for the five biogeographical regions of Switzerland (see Figure S6) included in the breeding distribution model in alphabetic order. Figure S6: Map of the biogeographic regions of Switzerland. Table S1: Strata for selecting the sampling locations for the abundance models with the number of 500 × 500 m squares (25 ha) randomly sampled in each stratum. Table S2: Parameter estimates, with standard error (SE) and 95% confidence intervals (CI) of the multinomial N‐mixture model used to predict Wallcreeper abundance for the overwintering period. Table S3: Parameter estimates, with standard error (SE) and 95% confidence intervals (CI), of the N‐mixture model used to predict abundance for the breeding period. Table S4: The three different metrics to assess model goodness of fit of multinomial N‐mixture models provided in the unmarked package, calculated using a parametric bootstrap with the function parboot separately for winter and the breeding modelsSee Kéry and Royle (2016) for details. Table S5: Table for the winter period showing a comparison between the curr [file ECE3-16-e73963-s001.docx]

**Supplementary Methods**

**Methods S1. Source of the Wallcreeper Occurrence Data and Methods of Data Selection for Analysis.** Wallcreeper occurrence data were extracted from the Swiss Ornithological Institute (SOI) database containing all observations of the species across Switzerland. This constituted N = 18,148 observations collected between 1 June 1909 and 31 August 2022. As only few absence data were recorded and these were not reliable due to the low detectability of the species, only presence locations were retained. For the wintering period, only observations between November and February were selected. For the breeding period, all observations between May and mid-July with the addition of the observations with an Atlas code equal to or greater than 3 (corresponding to a territorial singing individual) between April and August were extracted from the SOI database.

Most of the data were collected by volunteer observers on an opportunistic basis. In general, observers reported their observations with different levels of geographical accuracy using the *ornitho.ch* platform (*ornitho.ch* website or *NaturaList* smartphone app) or by sending them to the SOI. For a more systematically sampling of some species of interest, including the Wallcreeper, most of observers were asked by the SOI to report all their sightings of these target species during their outings. In some rare cases, presence data also came from the monitoring of common breeding birds in Switzerland (MHB) including the Biodiversity Monitoring Switzerland for birds (BDM), and Atlas surveys between 2013-2016, both following a systematic sampling of 1 km^2^ squares across Switzerland (Knaus et al., 2018; Locher & Van Wezemael, 2014; Schmid et al., 2004).

The data varied in locational accuracy, ranging from 10 km^2^ to 1 m^2^. This last very precisely localized data type was the result of observations entered via a map. Only data with high location accuracy (≤100 m, N = 2,310 for wintering, N = 1,846 for breeding) were retained, represented mainly by observations with an accuracy of 1 m^2^ and unknown real margin of error, given that it depends on the accuracy with which the observer entered it on the map. Respectively, 75% and 85% of these precise observations intersected the study area (i.e. on the rock face and their 150 m buffer). Observations entered outside of the study area were likely from birds commuting from one rock face to another, from birds visiting buildings (especially outside the breeding period) or resulting from spatially inaccurate recordings of the observation by the observer.

In the next step, all data outside the study area were excluded. In addition, we also excluded Swiss observations collected at less than 125 m from the Swiss border to match the extent of the environmental predictors (see Environmental Predictor Variables part in the main text). We then restricted the dataset to data from 2010 to August 2022, the period during which most of the data were collected. We did so in order to have only data obtained under contemporary climatic conditions and similar observation habits with the increasingly widespread use of the *ornitho.ch* platform and *NaturaList* smartphone app as a way to report observations.

**Methods S2. Field Sampling Protocol for Abundances.** The probability of detecting a Wallcreeper at a given site is very low, typically corresponding to roughly one observation of a few minutes every two hours (Luisier, unpublished data). For this reason, our survey protocol was specifically designed to maximize detection, while minimizing observer bias and time expenditure, and was based on ten years of experience by the lead author (CL) in monitoring populations of the species within Switzerland (e.g. Luisier, 2022). Specifically, we carefully surveyed rock faces within sampling sites at positions that allowed unobstructed views, using visual and auditory cues. We spent an average of 45 min per ha of rock face, which is the amount of effort required to reliably detect active individuals based on our previous experience. All surveys were conducted by one observer (CL). We repeated this protocol for a total of three visits at each site (Luisier, 2022; Luisier et al., 2022). Each Wallcreeper observed was photographed to allow individual identification using individual plumage patterns based on wing spots (Luisier & Wildi, 2023; Václav, 2016). Because we were interested in territorial individuals, we excluded Wallcreepers seen in a survey square that were obviously not in their respective territory (e.g., passing for a few minutes due to behavioural interactions or foraging commutes between two different territories).

As the Wallcreeper is territorial both during the breeding and the wintering season and should stay in the same area during a given period, we could assume almost no spatial emigration (i.e. a quasi-closed population, except for mortality) with the reasons for potential non-detection limited to behaviour and observer bias. However, as the species remains poorly known and mortality can occur, we separated visits by at least ten days. Moreover, the species’ detectability is presumed to peak during the formation of the winter territory in mid-November, and then to be stable for the rest of the wintering period (Glutz von Blotzheim & Bauer, 1993; Löhrl, 1976). We therefore started fieldwork in mid-November, with the first visit at each site conducted between mid-November and end of December, the second between 31 December 2022 and 26 January 2023 and the last one from 27 January 2023 to the end of February 2023, with some rare exceptions due to bad weather or snow conditions.

For surveys during the breeding period, we conducted visits according to site accessibility and peak of behavioural detectability, which correlates mostly with snow melt and altitude. In general, we carried out two visits during the mating period from the end of April to mid-June. During this time, birds were easier to detect due to breeding displays. We completed a third visit during the chick-feeding period from June to August. We tried to avoid the incubation period because the species is hard to detect during that time, given that the female is incubating and the male is not always active (Glutz von Blotzheim & Bauer, 1993; Luisier et al., 2020). Six sites were surveyed entirely during the chick-feeding period due to inaccessibility until the end of June or mid-July. Surveys were started as soon as possible after dawn (Luisier, 2022).

**Methods S3. Reduction of the sampling bias for species distribution models**

**Overwintering Period.** Generally, observers were concentrated in low altitude areas, which ensures a high detectability of birds wintering at low elevation. As Wallcreepers were located at lower altitude during the overwintering period, most territories are likely to be detected, however the resulting presence records are clumped in high densities in the areas that are most popular for observers wanting to see this charismatic species. To remove these clumps, we thinned the data (Aiello-Lammens et al., 2015; Kramer-Schadt et al., 2013) with a minimum distance of 200 m between locations using the *spThin* package (Aiello-Lammens et al., 2015), resulting in 756 locations. This distance reflected biological knowledge and aimed at selecting only one observation per possible territory (Luisier et al. 2022). Background locations were randomly selected from the entire study area.

**Breeding Period.** During the breeding period, most territories are located at high elevations where the observation pressure is very low due to limited accessibility to human observers. Therefore, the sample of recorded presence locations was strongly biased towards the few breeders at low altitude, while high-altitude breeders had a higher probability to remain undetected. As the usual thinning procedure would not have been sufficient in this context, because it doesn’t take into account the very low observation pression at high altitude, we accounted for this bias by performing a targeted selection of the background locations which enabled accounting for observation pressure (Kramer-Schadt et al., 2013). We first selected one presence location per 25 x 25 m raster cell to avoid duplicates and reduce spatial clumping, resulting in 1’497 locations. Using the package *SDMflex* the background locations (see modelling process with Maxent software) were then drawn in a number proportional to the values of a bias file (Velazco et al., 2022), which quantified observation pressure and was constructed using a target group approach (Barber et al., 2022; Kramer-Schadt et al., 2013).

As an estimate of spatial variation of observation pressure we used the number of days between April and August from 2010 to 2022 with at least one observation of Wallcreeper or of at least one of seven other species of birds known to often occur with the Wallcreeper in the same rock face as a target group (Common Kestrel, Crag Martin, Grey Wagtail, Alpine Accentor, Black Redstart, Alpine Chough, White-winged Snowfinch ). These data were provided by the SOI at a 1-km square resolution. To ensure that background data were drawn from the entire study area, we attributed a minimal value of two observation days to all squares with one or no days with observations. This minimal value was based on Luisier, (2022) showing, that for a random observer, it takes a median of two days with an observation of a target group species to make a first Wallcreeper observation in an occupied Wallcreeper breeding site (n = 21 sites).

**Methods S4. Environmental Predictors.** The variability of exposition was calculated using the standard deviation of the sine of the aspect from the digital elevation model. The proportion of rock within 1 km was calculated to account for the suitability of the local habitat at a larger scale given that Wallcreepers require rock faces for both breeding and wintering habitat. The average seasonal solar radiation was calculated using ArcMap version 10.8.2 as the average daily solar radiation between 1 December 2013 and 28 February 2014 for the overwintering period and between 1 May 2014 and 31 July 2014 for the breeding period to focus on the physiologically more demanding period and match with the temporal resolution of the other climatic predictors. To produce a predictor for gorges, which are a suitable habitat type for the species, a buffer of 50 m was applied around all rivers that are present year- round (swissTLM3D) and then intersected with the Swiss rock faces (swissTLM3D). The resulting shapefile was then converted to a raster using the same extent and cell size as the other predictors before applying the moving window. Finally, the two connectivity metrics were obtained using functions from the *grainscape* package (Chubaty et al., 2020). We first extracted the minimum planar graph of the Swiss rock faces using the MPG function, setting the resistance for rock faces to 0 as suitable patches and the other areas to 1 as being unsuitable. The MPG is spatial network that connects the rock faces using the shortest possible paths, while ensuring that no connections cross each other. For the small-scale connectivity between rock faces that are near to each other, we applied the degree function to the MPG output to assess it for each node (i.e. rock face). It quantifies how directly connected each rock face is to its neighbours. For the links to centroids, we used the links to centroids map from the MPG output and smoothed the links with the line density function at a 7’308 m distance suggested by ArcMap version 10.8.2. It quantifies the connection importance of the rock face in the global landscape. All other predictors were obtained directly from different institutes, mostly as vector maps (Table 1).

**Methods S5: Selection criteria for correlated environmental predictors.** The table below lists pairs of correlated predictors (Spearman’s |rS| > 0.7) with pair numbers and correlation values. For each pair exceeding this threshold, the predictor considered biologically least important for the Wallcreeper was removed, based on literature and expert knowledge. This is explained for each pair below the table, with the selected predictors underlined.

| **Pair** | **Predictor 1** | **Predictor 2** | **Correlation coefficient** |
| --- | --- | --- | --- |
| 1 | Elevation | Average seasonal ambient temperature | -0.99 |
| 2 | Forest distance | Average seasonal ambient temperature | -0.87 |
| 3 | Northness | Average seasonal solar radiation | -0.87 |
| 4 | Elevation | Forest distance | 0.87 |
| 5 | Distance to the next forest | Forest frequency | -0.87 |
| 6 | Distance to the next grassland | Grassland and unproductive vegetation frequency | -0.84 |
| 7 | Forest cover | Average seasonal ambient temperature | 0.77 |
| 8 | Elevation | Forest frequency | -0.77 |

1: More direct abiotic effect due to the temperature.

2: Temperature has a direct effect on the whole habitat, whereas forest has just an indirect effect on food resources (Glutz von Blotzheim & Bauer, 1993; Luisier et al., 2020).

3: Solar radiation has a direct effect on the whole habitat, whereas northness has just an indirect effect on food resources and breeding sites availability (Glutz von Blotzheim & Bauer, 1993; Luisier et al., 2020).

4: Both already excluded.

5: Both already excluded.

6: Grasslands produce prey that must ultimately move towards cliff habitats (Glutz von Blotzheim & Bauer, 1993; Luisier et al., 2020). As most of these prey are flying insects, they can originate from some distance; however, their dispersal is not unlimited. The frequency therefore reflects local prey production in grasslands at a fine spatial scale, which is likely less influential than prey inputs coming from a certain limited distance.

7: Temperature has a direct effect on the whole habitat, whereas forest has just an indirect effect on food resources (Glutz von Blotzheim & Bauer, 1993; Luisier et al., 2020).

8: Both already excluded.

**Supplementary Tables and Figures**

Table S1: Strata for selecting the sampling locations for the abundance models with the number of 500 x 500 m squares (25 ha) randomly sampled in each stratum. The occurrence probability intervals represent the mean predicted occurrence probability of Wallcreeper at the rock faces in each square from the distribution model with four strata (< 0.25, 0.25 - 0.50, 0.50 - 0.75 and > 0.75). The rock face area is represented as the number of raster cells considered as rock face in the distribution model, with a maximum number of 400 making 25 ha.

| Occurrence probability | Rock face area (raster cells) | Number of squares selected | |
| --- | --- | --- | --- |
|  |  | Overwintering | Breeding |
| 0 - 0.25 | 32 - 200 | 2 | 4 |
|  | 201 - 400 | 2 | 4 |
| 0.25 - 0.50 | 32 - 200 | 2 | 4 |
|  | 201 - 400 | 2 | 4 |
| 0.50 - 0.75 | 32 - 200 | 4 | 4 |
|  | 201 - 400 | 2 | 4 |
| 0.75 - 1 | 32 - 200 | 2 | 4 |
|  | 201 - 400 | 2 | 4 |
| Total |  | 18 | 32 |

Table S2: Parameter estimates, with standard error (SE) and 95% confidence intervals (CI) of the multinomial N-mixture model used to predict Wallcreeper abundance for the overwintering period. The abundance parameters are on the log-scale and the detection probability parameters are on the logit-scale. “Mean occurrence probability” is the species’ mean occurrence probability in the 500 x 500m square predicted by the distribution model, “Visit” is the visit number (1, 2 or 3), “Sun” the proportion of rock face exposed to the sun during the visit (0 to 1) and “temperature” the mean temperature during the visit in degrees Celsius.

| **Model parameters** | **Estimate** | **SE** | **95% CI** |
| --- | --- | --- | --- |
| Abundance (λ) |  | | |
| Intercept | -1.200 | 0.578 | -2.330 – -0.063 |
| Mean occurrence probability | 4.250 | 1.443 | 1.425 – 7.083 |
| Detection probability (p) |  | | |
| Intercept | -0.387 | 0.430 | -1.229 – 0.456 |

Table S3: Parameter estimates, with standard error (SE) and 95% confidence intervals (CI), of the N-mixture model used to predict abundance for the breeding period. The abundance parameters are on the log-scale and the detection probability parameters are on the logit-scale. “Mean occurrence probability” is the species’ mean occurrence probability in the 500 x 500m square predicted by the distribution model, “Visit” is the visit number (1, 2 or 3), “Sun” the proportion of rock face exposed to the sun during the visit (0 to 1) , “temperature” the mean temperature during the visit in degrees Celsius and “Days before fledge” the number of days before fledglings are expected to leave the territory (e.g., 10 days after the estimated fledge).

| **Model parameters** | **Estimate** | **SE** | **95% CI** |
| --- | --- | --- | --- |
| Abundance (λ) |  | | |
| Intercept | -1.66 | 0.513 | -5.058 – -0.626 |
| Mean occurrence probability | 3.58 | 1.292 | 1.051 – 6.116 |
| Detection probability (p) |  | | |
| Intercept | 1.86 | 1.08 | -0.262 – 3.974 |
| Visit 2 | -2.84­­­­ | 1.13 | -5.058 – -0.626 |
| Visit 3 | -2.51 | 1.12 | -4.713 – -0.303 |
| Sun | 2.08 | 1.46 | -0.790 – -4.951 |

Table S4: The three different metrics to assess model goodness of fit of multinomial N-mixture models provided in the unmarked package , calculated using a parametric bootstrap with the function parboot separately for winter and the breeding modelsSee Kéry & Royle (2016) for details

| **Method** | **Mean** | **95% CI** | **P-value** |
| --- | --- | --- | --- |
| *Overwintering* |  |  |  |
| Chisq | 100.3 | 67.3-178 | 0.865 |
| SSE | 10.8 | 5.3-22 | 0.608 |
| FT | 15.4 | 7.7-3.22 | 0.471 |
| *Breeding* |  |  |  |
| Chisq | 191.2 | 74.3-418 | 0.303 |
| SSE | 14.7 | 7.8-24 | 0.502 |
| FT | 19.4 | 10.9-26 | 0.388 |

Table S5: Table for the winter period showing a comparison between the current distribution prediction 2010-2022 and the four different future distribution predictions (i.e. short term time interval 2030-2050 and long term time interval 2080-2100 each under moderate greenhouse gas emission rcp45 scenario and extreme greenhouse gas emission scenario rcp85) with the number of suitable cells, the projected suitable area (km^2^), the percentage of the total area and the percentage of difference since 2010-2022. The projected suitable area is calculated by taking into account that each cell of 25 x 25 m has an area of 625 m^2^, which is an underestimation because it does not take into account the slope and all the small elements of the rock faces which make it more complex. See also Fig. 3.

| **Prediction** | **Number of suitable cells** | **Projected suitable area (km^2^)** | **Percentage of the total area** | **Percentage of variation since 2010-2022** |
| --- | --- | --- | --- | --- |
| ***2010-2022*** | 1,217,574 | 761 | 14.4 | 0 |
| ***2030-2050 rcp45*** | 1,862,217 | 1,164 | 23.6 | +53 |
| ***2030-2050 rcp85*** | 2,129,101 | 1,331 | 27 | +74.9 |
| ***2080-2100 rcp45*** | 2,666’546 | 1,667 | 33.8 | +119 |
| ***2080-2100 rcp85*** | 4,186,262 | 2,616 | 53 | +243.8 |

Table S6: Comparison between the prediction of the current breeding distribution 2010-2022 and the four different future breeding distribution predictions (i.e. short term time interval 2030-2050 and long term time interval 2080-2100 each under moderate greenhouse gas emission rcp45 scenario and extreme greenhouse gas emission scenario rcp85), with the number of suitable cells, the projected suitable area (km^2^), the percentage of the total area and the percentage of variation since 2010-2022. The projected suitable area is calculated by taking into account that each cell of 25x25 m has an area of 625 m^2^, which is an underestimation because it does not take into account the slope and the actual area the complex rock surfaces. See also Fig. 3.

| **Prediction** | **Number of suitable cells** | **Projected suitable area (km^2^)** | **Percentage of the total area** | **Percentage of variation since 2010-2022** |
| --- | --- | --- | --- | --- |
| ***2010-2022*** | 3,063,485 | 1,915 | 38.9 | 0 |
| ***2030-2050 rcp45*** | 3,007,006 | 1,879 | 38.2 | -1.9 |
| ***2030-2050 rcp85*** | 3,198,434 | 1,999 | 40.6 | +4.4 |
| ***2080-2100 rcp45*** | 3,194,996 | 1,997 | 40.6 | +4.3 |
| ***2080-2100 rcp85*** | 3,080,240 | 1,925 | 39.1 | +0.5 |


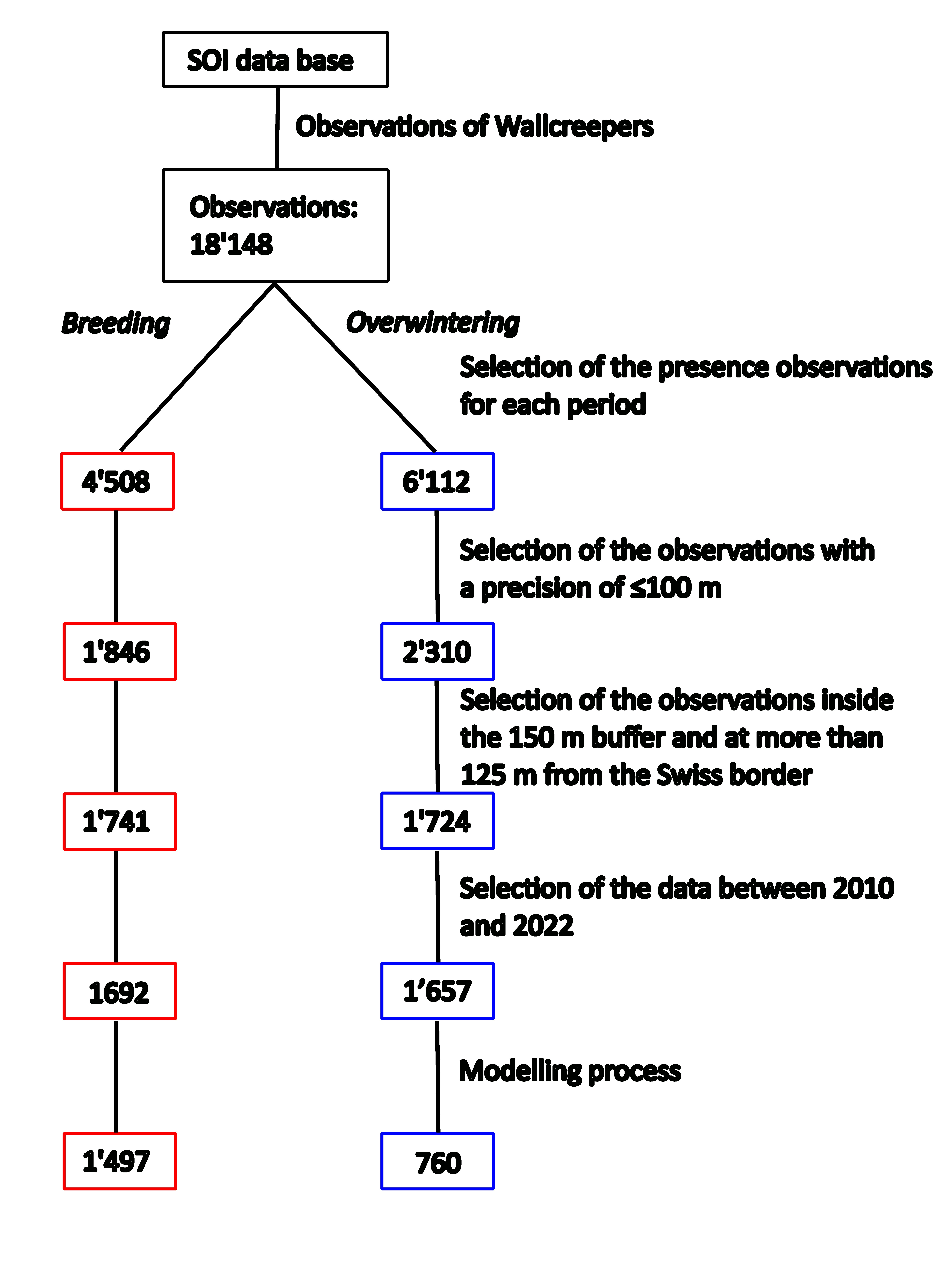


Figure S1: Flowchart presenting the selection process of Wallcreeper presence data and number of locations retained after each filtering step for the breeding (red) and the overwintering (blue) periods. Filtering steps are the same for both periods and described on the very right. Observations were selected inside a 150 m buffer around all Swiss rock faces. All the data were extracted from the database at the Swiss Ornithological Institute (SOI).


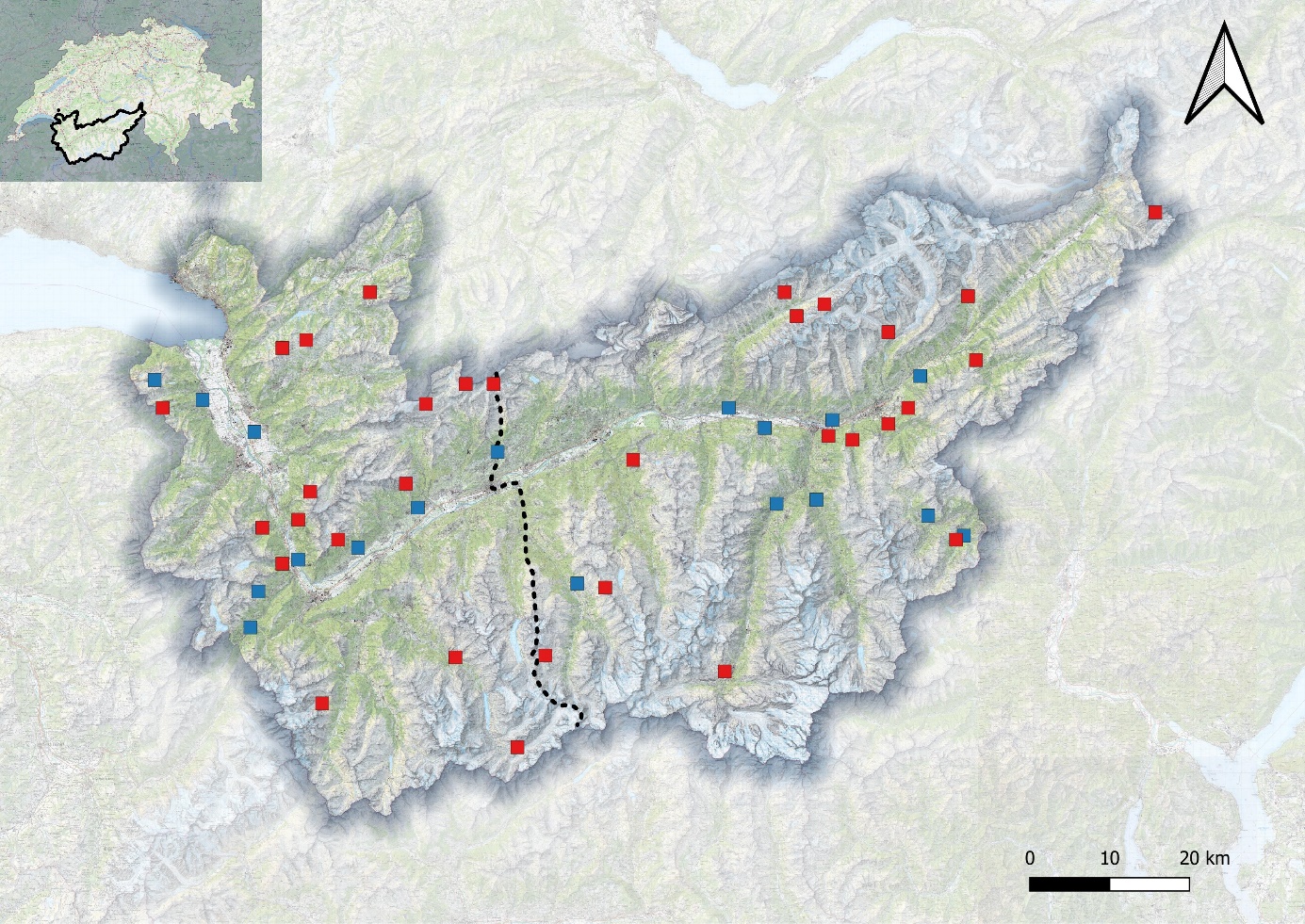


Figure S2: Abundance sampling sites for the overwintering (blue) and breeding (red) periods in the abundance study area and location of the study area in black within Switzerland (top left). The dotted line follows the separation between the two parts of the study area used to spatially balance the sampling.

*
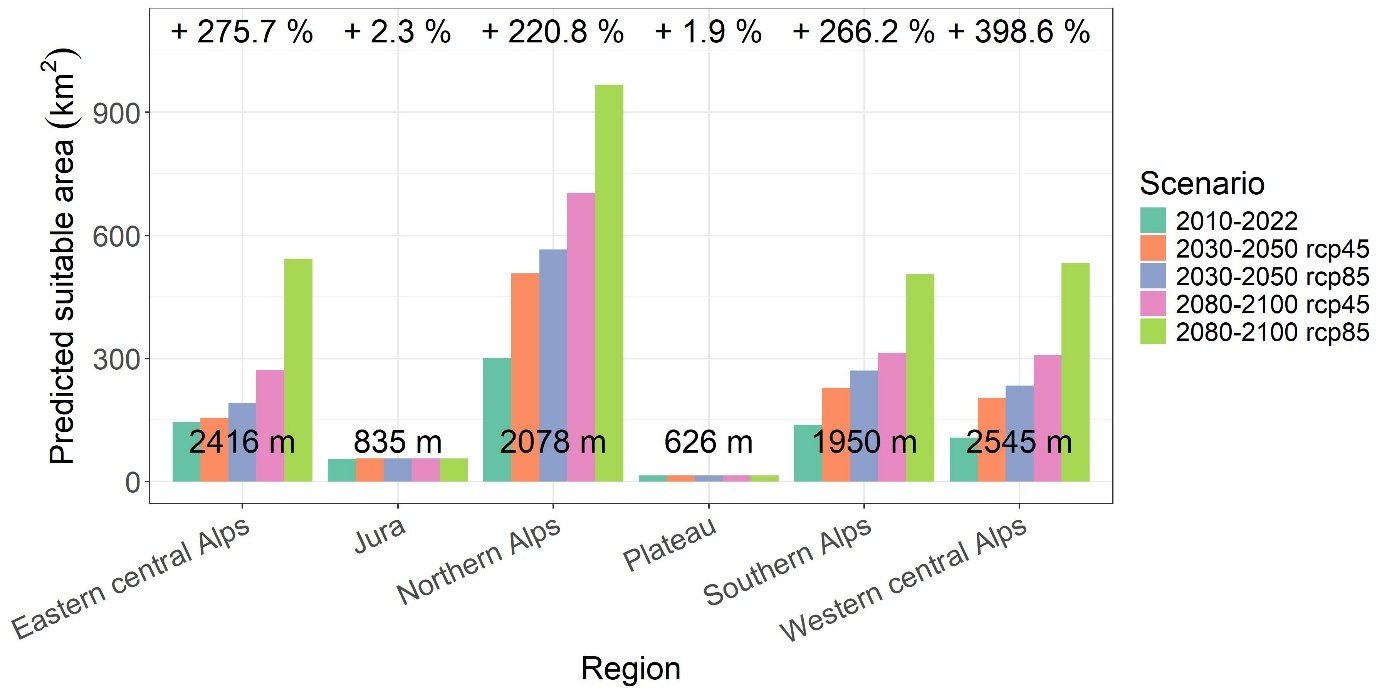
*

Figure S3: Predicted area of Wallcreeper winter distribution for the six biogeographical regions of Switzerland included in the overwintering distribution model in alphabetic order. In each group of bars, the current (2010-2022) and the four future projections (i.e. 2030-2050 rcp45 and rcp85 and 2080-2100 rcp45 and rcp85) are presented. The number in each group of bar represents the mean altitude of the rock faces in the region and the number at the top the percentage of difference in predicted distribution area between the current and the 2080-2100 rcp85 (most extreme one) projections. See Figure S6 for a map of the biogeographic regions.

(A)


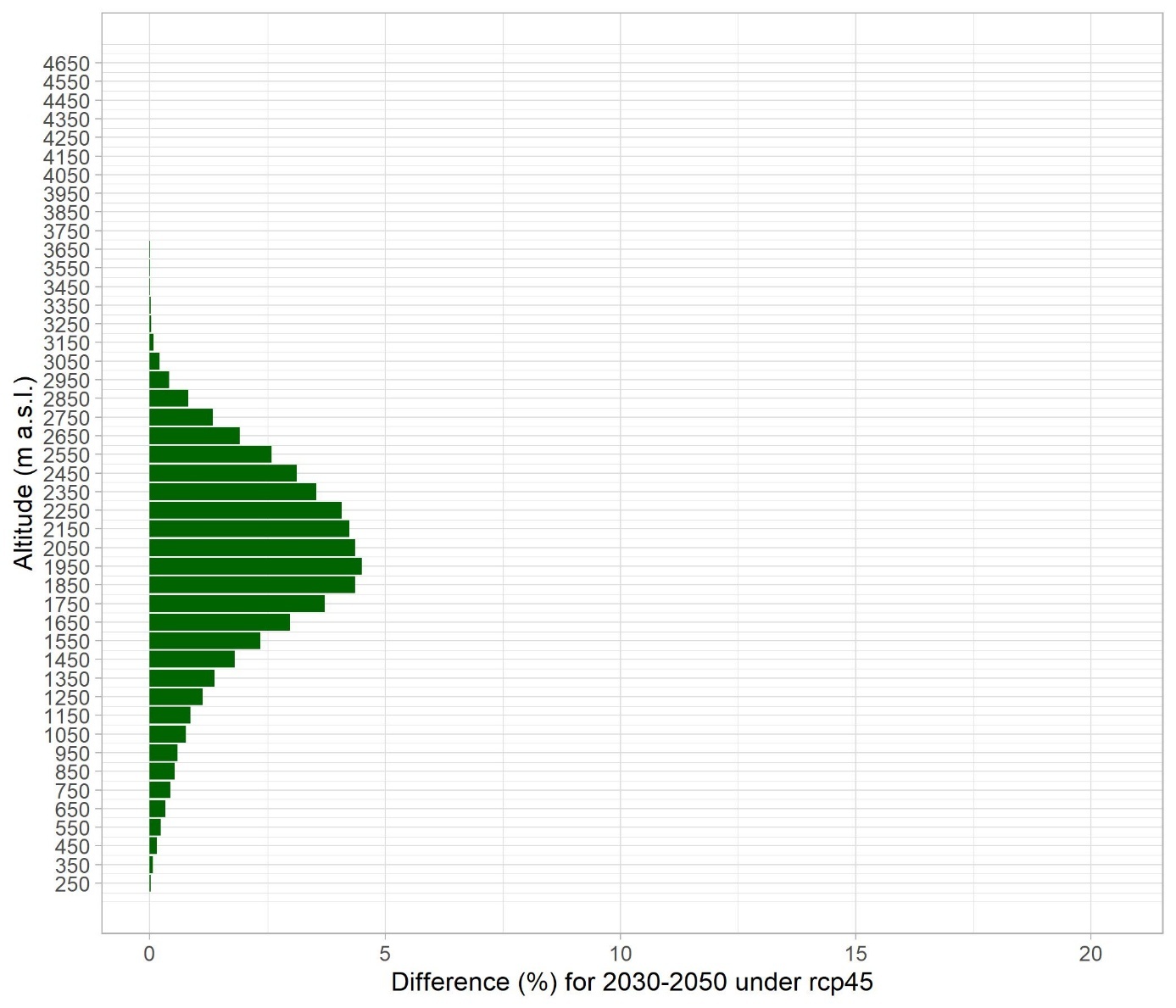


(B)


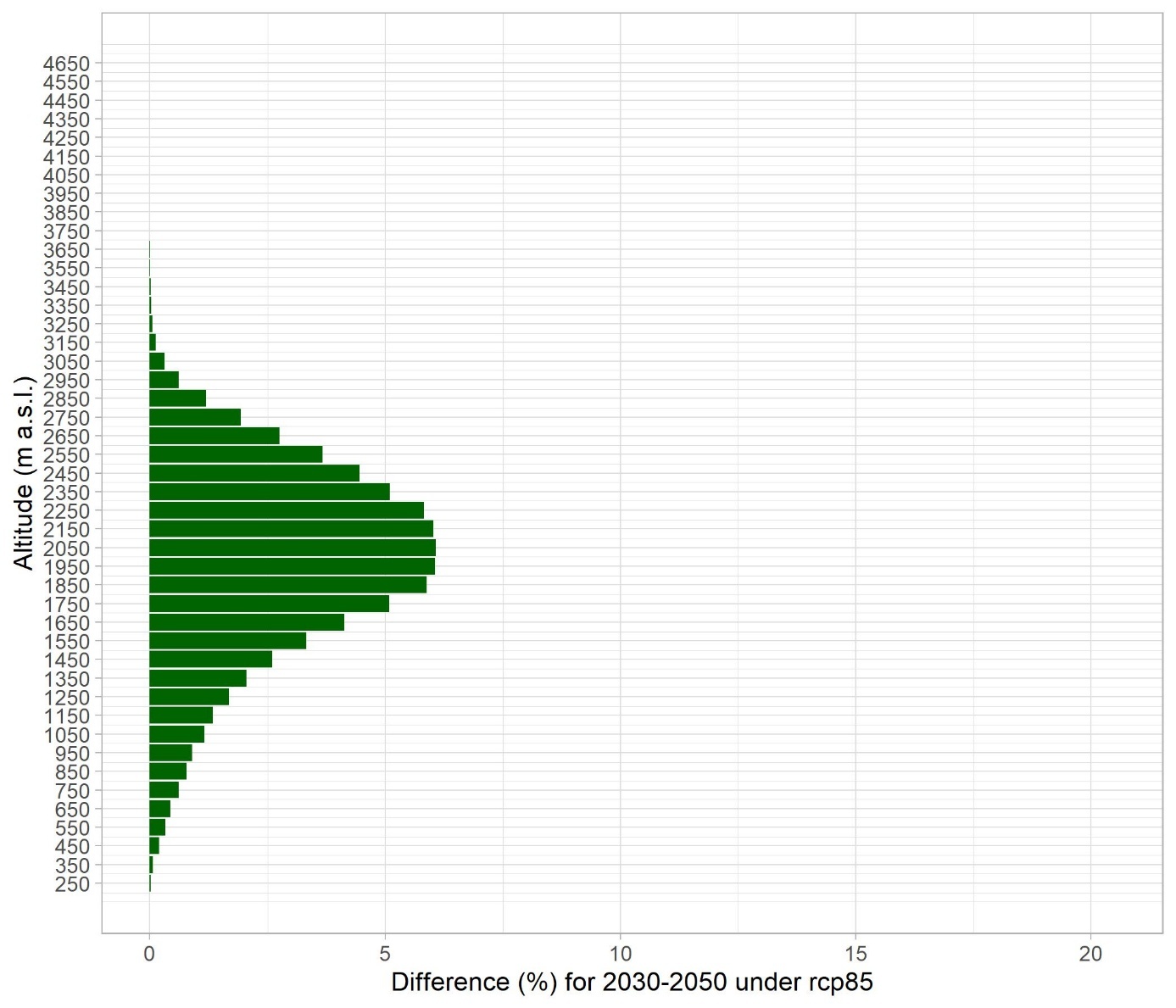


(C)


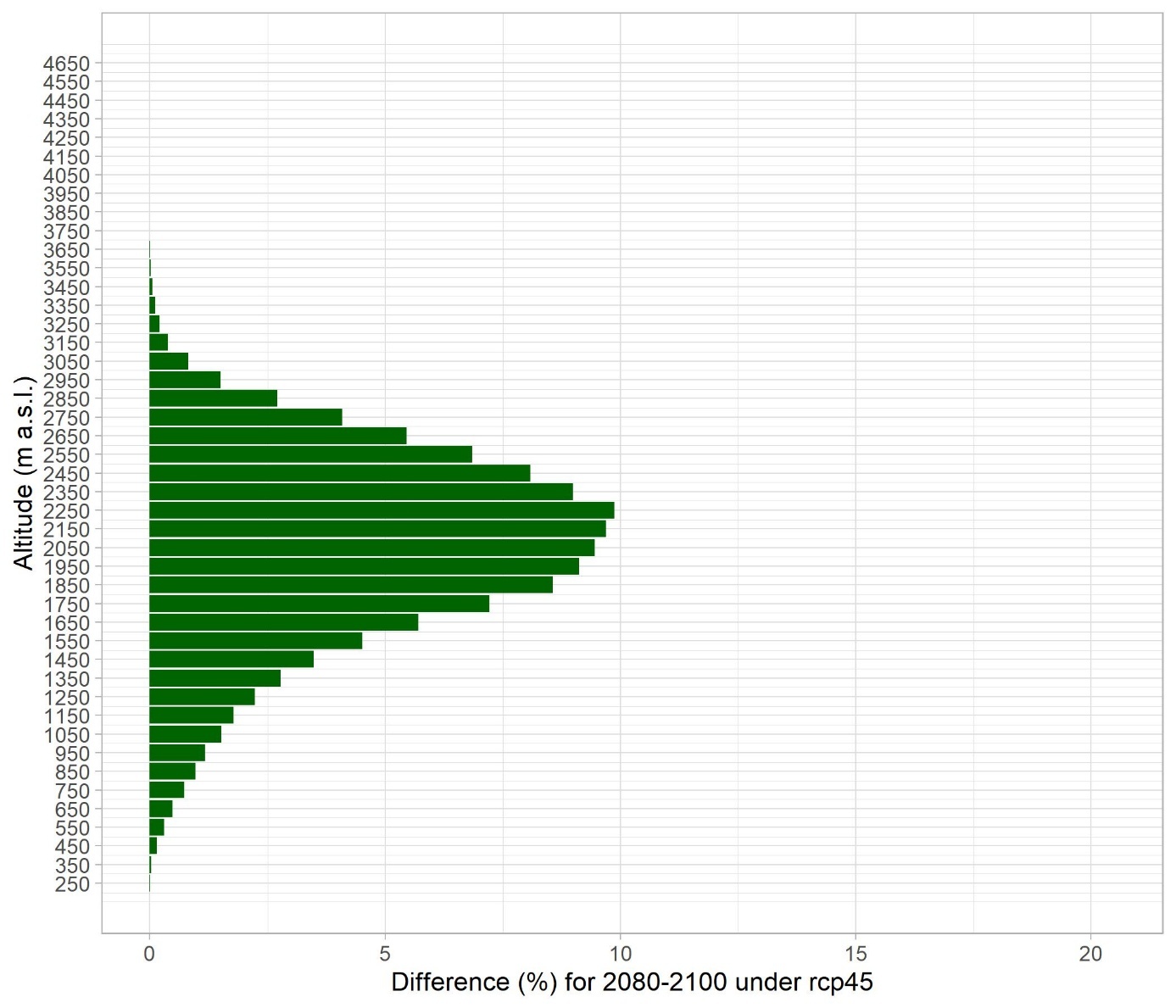


(D)


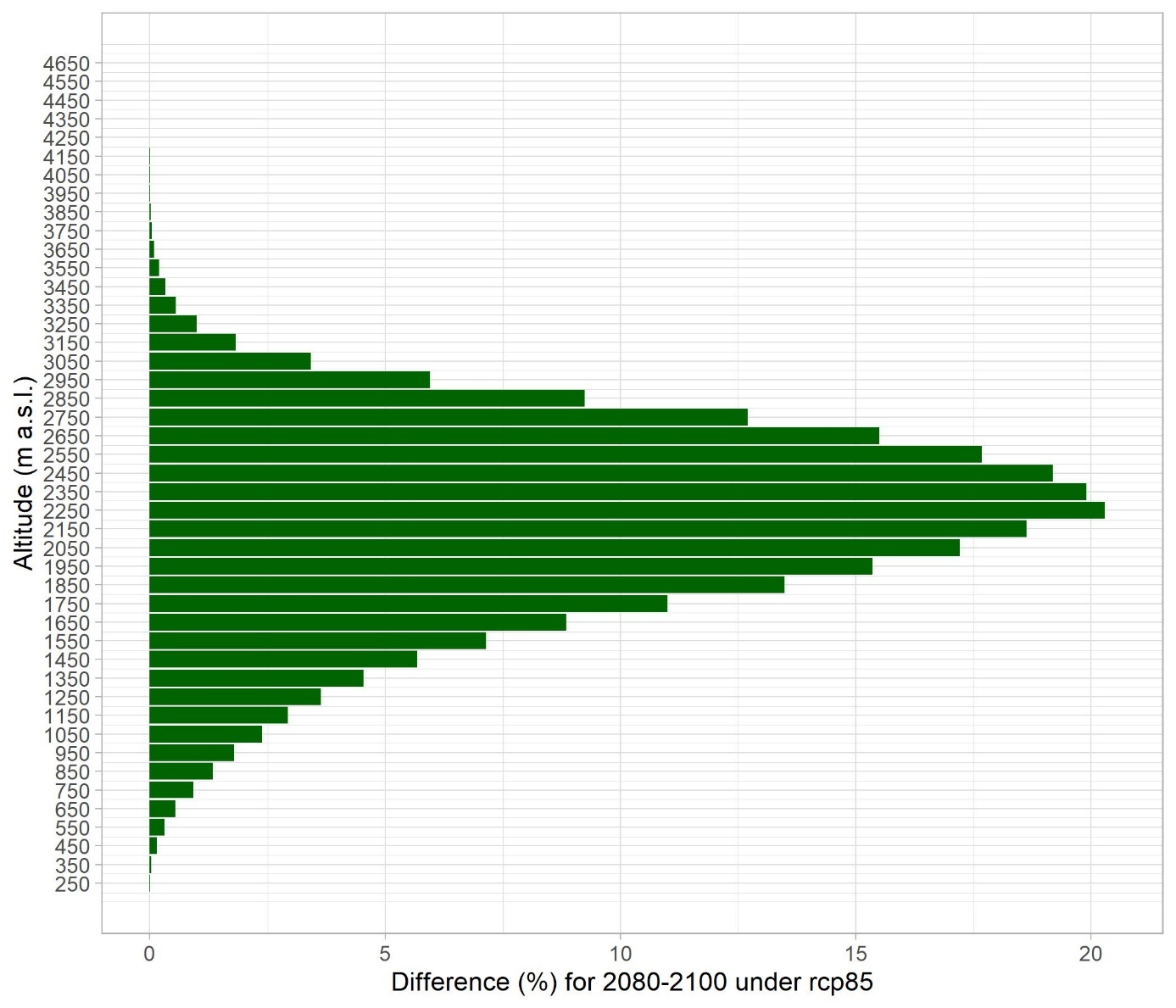


Figure S4: Percent difference in Wallcreeper overwintering area (i.e. presence cells) between the current four future predictions (2030-2050 under rcp45 (A) and rcp85 (B) and 2080-2100 under rcp45 (C) and rcp85 (D), calculated for altitude bands of 100 m based on the species distribution model. See legend of Fig. 2 for more details about climate scenarios. The green bars represent the gains, whereas there are overall no losses inside of each band.

*
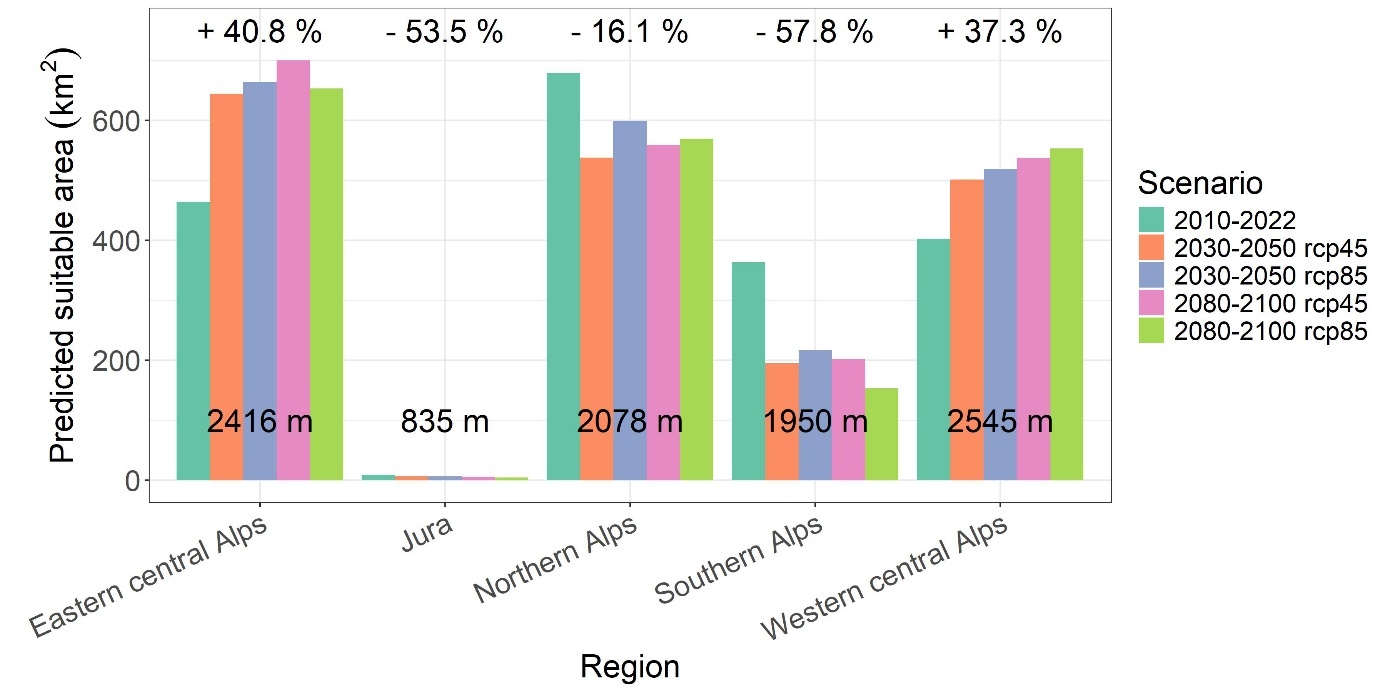
*

Figure S5: Predicted area of Wallcreeper distribution for the five biogeographical regions of Switzerland (see Figure S6) included in the breeding distribution model in alphabetic order. The Plateau was excluded because the Wallcreeper doesn’t breed outside of the mountains. In each group of bars, the current and the four future projections are presented (i.e. 2010-2022, 2030-2050 rcp45 and rcp85 and 2080-2100 rcp45 and rcp85; see Fig. 1 for more details). The number in each group of bar represents the mean altitude of the rock faces in the region and the number at the top the percentage of difference in predicted distribution area between the current and the 2080-2100 rcp85 (most extreme one) projections. See Figure S6 for map of biogeographic regions.


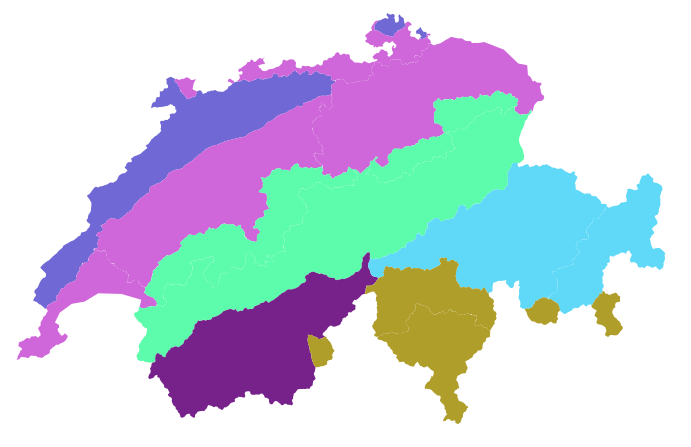

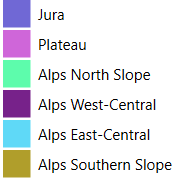

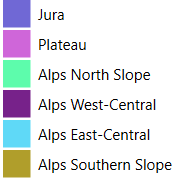


Figure S6. Map of the biogeographic regions of Switzerland.

**Supplementary References**

Aiello-Lammens, M. E., Boria, R. A., Radosavljevic, A., Vilela, B., & Anderson, R. P. (2015). spThin: An R package for spatial thinning of species occurrence records for use in ecological niche models. *Ecography*, *38*(5), 541–545. https://doi.org/10.1111/ecog.01132

Barber, R. A., Ball, S. G., Morris, R. K. A., & Gilbert, F. (2022). Target-group backgrounds prove effective at correcting sampling bias in Maxent models. *Diversity and Distributions*, *28*(1), 128–141. https://doi.org/10.1111/ddi.13442

Chubaty, A. M., Galpern, P., & Doctolero, S. C. (2020). The r toolbox grainscape for modelling and visualizing landscape connectivity using spatially explicit networks. *Methods in Ecology and Evolution*, *11*(4), 591–594. https://doi.org/10.1111/2041-210X.13350

Kéry, M., & Royle, J. A. (2016). Chapter 7 - Modeling abundance using multinomial *N*-mixture models. In M. Kéry, & J. A. Royle (Eds.), Applied hierarchical modeling in ecology (pp. 313–392). Academic Press.

Knaus, P., Antonniazza, S., Wechsler, S., Guélat, J., Kéry, M., Strebel, N., & Sattler, T. (2018). *Atlas des oiseaux nicheurs de Suisse 2013-2016. Distribution et évolution des effectifs des oiseaux en Suisse et au Liechtenstein.* Swiss ornithological institute.

Kramer-Schadt, S., Niedballa, J., Pilgrim, J. D., Schröder, B., Lindenborn, J., Reinfelder, V., Stillfried, M., Heckmann, I., Scharf, A. K., Augeri, D. M., Cheyne, S. M., Hearn, A. J., Ross, J., Macdonald, D. W., Mathai, J., Eaton, J., Marshall, A. J., Semiadi, G., Rustam, R., … Wilting, A. (2013). The importance of correcting for sampling bias in MaxEnt species distribution models. *Diversity and Distributions*, *19*(11), 1366–1379. https://doi.org/10.1111/ddi.12096

Locher, S., & Van Wezemael. (2014). Rapport méthodologique du MBD Description des méthodes et indicateurs. *Connaissance de l’environnement*, *1410*. www.bafu.admin.ch/uw-1410-f

Luisier, C. (2022). *Suivi du Tichodrome échelette (Tichodroma muraria) dans l’ouest des Alpes suisses : rapport 2022*. <https://doi.org/10.13140/RG.2.2.30695.87206>

Luisier, C., & Wildi, J. (2023). Individual identification of Wallcreepers in the field: how far can you go? Dutch Birding, 43(3), 190–194

Luisier, C., Wildi, J., & Saino Calabretta, M. (2022). Hivernage du Tichodrome échelette *tichodroma muraria* sur six grands complexes rocheux dans le sud-ouest de la Suisse : densités, chevauchement de territoires et alimentation. *Alauda*, *90*(3), 183–192.

Pazúr, R., Huber, N., Weber, D., Ginzler, C., & Price, B. (2022). A national extent map of cropland and grassland for Switzerland based on Sentinel-2 data. *Earth System Science Data, 14*(1), 295–305. <https://doi.org/10.5194/essd-14-295-2022>

Schmid, H., Zbinden, N., & Keller, V. (2004). *Überwachung der Bestandsentwicklung häufiger Brutvögel in der Schweiz*. Swiss ornithological institute.

Velazco, S. J. E., Rose, M. B., de Andrade, A. F. A., Minoli, I., & Franklin, J. (2022). flexsdm: An r package for supporting a comprehensive and flexible species distribution modelling workflow. *Methods in Ecology and Evolution*, *13*(8), 1661–1669. https://doi.org/10.1111/2041-210X.13874
